# The role of gene fusions in the evolution of metabolic pathways: the histidine biosynthesis case

**DOI:** 10.1186/1471-2148-7-S2-S4

**Published:** 2007-08-16

**Authors:** Renato Fani, Matteo Brilli, Marco Fondi, Pietro Lió

**Affiliations:** 1 Dept. of Animal Biology and Genetics, via Romana 17, 50125 Florence, Italy; 2 Computer Laboratory, University of Cambridge, CB3 0FD, Cambridge, UK

## Abstract

**Background:**

Histidine biosynthesis is one of the best characterized anabolic pathways. There is a large body of genetic and biochemical information available, including operon structure, gene expression, and increasingly larger sequence databases. For over forty years this pathway has been the subject of extensive studies, mainly in *Escherichia coli *and *Salmonella enterica*, in both of which details of histidine biosynthesis appear to be identical. In these two enterobacteria the pathway is unbranched, includes a number of unusual reactions, and consists of nine intermediates; *his *genes are arranged in a compact operon (*hisGDC [NB]HAF [IE]*), with three of them (*hisNB*, *hisD *and *hisIE*) coding for bifunctional enzymes. We performed a detailed analysis of *his *gene fusions in available genomes to understand the role of gene fusions in shaping this pathway.

**Results:**

The analysis of HisA structures revealed that several gene elongation events are at the root of this protein family: internal duplication have been identified by structural superposition of the modules composing the TIM-barrel protein.

Several *his *gene fusions happened in distinct taxonomic lineages; *hisNB *originated within *γ*-proteobacteria and after its appearance it was transferred to *Campylobacter *species (*ε*-proteobacteria) and to some Bacteria belonging to the CFB group. The transfer involved the entire *his *operon. The *hisIE *gene fusion was found in several taxonomic lineages and our results suggest that it probably happened several times in distinct lineages.

Gene fusions involving *hisIE *and *hisD *genes (*HIS4*) and *hisH *and *hisF *genes (*HIS7*) took place in the Eukarya domain; the latter has been transferred to some *δ*-proteobacteria.

**Conclusion:**

Gene duplication is the most widely known mechanism responsible for the origin and evolution of metabolic pathways; however, several other mechanisms might concur in the process of pathway assembly and gene fusion appeared to be one of the most important and common.

## Background

**Figure 1:**
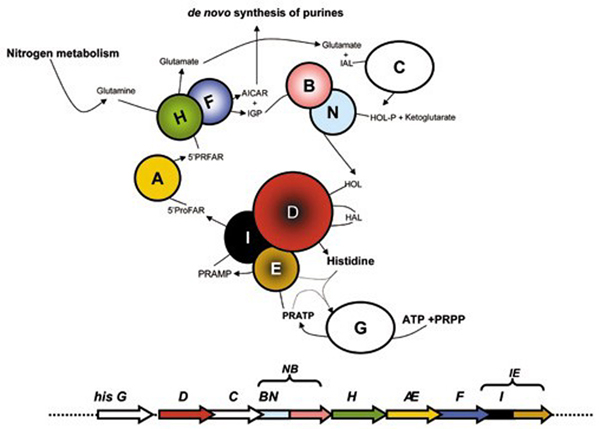
**Summary of Histidine biosynthesis**. Schematic representation of the histidine biosynthetic pathway and the organization of *his *gene in *Escherichia coli*. Genes and proteins in color are those involved in fusion events.

Histidine biosynthesis is one of the best characterized anabolic pathways. There is a large body of genetic and biochemical information available, mainly for *Escherichia coli *and *Salmonella enterica*, including operon structure, gene expression, and growing sequence data [1]. In these two enterobacteria, the pathway is the same, unbranched, includes a number of unusual reactions, and consists of nine intermediates; *his *genes are arranged in a compact operon (*hisGDC [NB]HAF [IE]*), with three of them (*hisNB*, *hisD *and *hisIE*) coding for bifunctional enzymes (Figure 1) [2,3].

Histidine biosynthesis is a metabolic cross-road and plays an important role in cellular metabolism being interconnected to both the *de novo *synthesis of purines and to nitrogen metabolism. The connection to purine biosynthesis results from an enzymatic step catalyzed by imidazole glycerol phosphate (IGP) synthase, a heterodimeric protein composed by one subunit each of the *hisH *and *hisF *products [2]. This heterodimeric enzyme catalyzes the transformation of N'-(5'-phosphoribosyl)-formimino-5-aminoimidazol-4-carboxamide ribonucleotide (PRFAR) into 5'-(5-aminoimidazole-4-carboxamide) ribonucleotide (AICAR), which is recycled into the *de novo *purine biosynthetic pathway, and IGP, which leads to histidine. The important connection to nitrogen metabolism is due to a glutamine molecule, the source of the final nitrogen atom of the imidazole ring of IGP. Chemical and biological evidences suggest that histidine was formed during the long period of chemical abiotic synthesis of organic compounds and the monophyly of the three cell domains in phylogenetic trees of concatenated His proteins, suggests that this biosynthetic route is ancient. The chemical syntheses of histidine [4], prebiotic analogues of histidine [5], and of histidyl-histidine under primitive conditions has been reported [6], as well as the role of the latter in the enhancement of some possible prebiotic oligomerization reactions involving amino acids [7] and nucleotides [8]. It is therefore reasonable to assume that His-containing small peptides could have been involved in the prebiotic formation of other peptides and nucleic acid molecules, once these monomers accumulated in primitive tidal lagoons or ponds. If primitive catalysts required histidine, then the eventual exhaustion of the prebiotic supply of histidine and histidine-containing peptides [4,6,8] imposed a selective pressure favoring those organisms capable of synthesizing histidine. Hence this metabolic pathway might have been assembled long before the appearance of the Last Universal Common Ancestor (LUCA) and the wet-lab and bioinformatics work carried out by our group in the last 15 years strongly supported this thesis [2,9-14]. A wide variety of clustering strategies of *his *genes have been documented [10]; moreover, an impressive series of well characterized duplication, elongation, and fusion events has shaped this pathway. Therefore, the histidine biosynthetic pathway represents a very good model for understanding the molecular mechanisms driving the assembly and refining of metabolic routes.

It is worth noting that at least seven genes, namely *hisD*, *hisB*, *hisN*, *hisI*, *hisE*, *hisF*, and *hisH *underwent fusion events in different phylogenetic lineages [11-13,15]. Gene fusions provide a mechanism for the physical association of different protein domains that might be catalytic or regulatory [16]. Moreover, fusions frequently involve genes coding for proteins that function in a concerted manner, such as enzymes catalyzing sequential steps within a metabolic pathway [17]. Fusion of such catalytic centers likely facilitates the channeling of intermediates [16]; the high fitness of gene fusions can also rely on the tight regulation of the expression of the fused domains.

Besides, a special case of gene fusion has played a key role in the evolution of ancestral genes: several proteins have been shown to be the outcome of coupled "duplication and fusion" events (gene elongation). The outcome of such an event is a gene with two paralogous moieties (modules) that might undergo further duplication events, leading to a gene with several internal repetitions. Gene elongation events allow improving a protein's function by increasing the number of active sites and/or the acquisition of an additional function by modifying a redundant segment. The most documented example pertains two *his *genes, *hisA *and *hisF*, encoding two (*β*/*α*)_8 _barrel (TIM-barrel) proteins [18].

The aim of this work is to evaluate the overall role that gene fusion(s) might have had in the context of the assembly and evolution of histidine biosynthetic route, and to understand the biological significance of each fusion. For this purpose, the structure and organization of all the available *his *genes that underwent fusion event(s) were analyzed using statistical and bioinformatics methods.

## Results and discussion

### A cascade of gene elongations and duplications: *hisA *and *hisF*

The two genes *hisA *and *hisF *code for a [N-(5'-phosphoribosyl) formimino]-5-aminoimidazole-4-carboxamide ribonucleotide (ProFAR) isomerase and a cyclase, respectively, which catalyze two central and sequential reactions (the fourth and fifth ones) of the pathway (Figure 1) and belong to the TIM-barrel family of proteins. The comparative analysis of the HisA and HisF proteins from different archaeal, bacterial, and eukaryotic (micro)organisms revealed that they are paralogous and share a similar internal organization into two paralogous modules half the size of the entire sequence [18]. Comparison of these modules led to the suggestion that *hisA *and *hisF *are the result of two ancient successive duplications, the first one involving an ancestral module half the size of the present-day *hisA *gene and leading (by a gene elongation event) to the ancestral *hisA *gene, which in turn underwent a duplication that gave rise to the ancestor of *hisF *[18].

The barrel structure is composed by eight concatenated (*β*-strand)-loop-(*α*-helix) units. The *β*-strands are located in the interior of the protein, forming the staves of a barrel, whereas the *α*-helices pack around them facing the exterior. According to the model proposed [18,19] the ancestral half-barrel gave a functional enzyme by homo-dimerization. The elongation event leading to the ancestor of *hisA*/*hisF *genes resulted in the covalent fusion of two half-barrels producing a protein whose function was refined and optimized by mutational changes; once assembled, the "whole-barrel gene" underwent gene duplication, leading to the ancestor of *hisA *and *hisF *[18,19].

**Figure 2:**
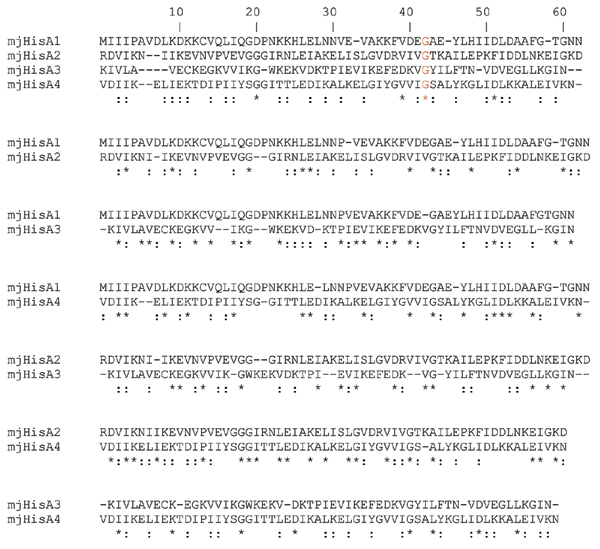
**HisA "quarters" alignments**. Pairwise alignments of *Methanocaldococcus jannaschii *(mj) HisA subregions corresponding to "quarters" of barrels (named HisA1, HisA2, HisA3, HisA4). Symbols: *,: correspond to identical or similar aminoacids, respectively.

**Figure 3:**
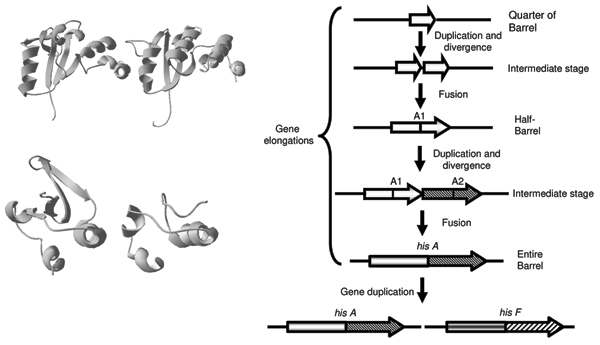
**An evolutionary model for *hisA *and *hisF *genes**. Right-most panel is the evolutionary model that we propose and discuss in the text concerning *hisA *and *hisF *origin and evolution. Panels on the left are: the first and the second quarters (top) and two single (*β*/*α*) modules of the HisA protein from *Thermotoga maritima *which illustrates the structural similarities from which we derived our model. The quarters have a structural alignment with only 1.2 Å of RMS on 104 alpha carbons.

The structural symmetry of the TIM barrel has prompted us to investigate the possibility of an even older gene elongation event involving (*β*/*α*)-mers smaller than the (*β*/*α*)_4 _units of the ancestral "half-barrel" precursor. To this purpose an extensive analysis of all the available HisA and HisF sequences was carried out. This analysis was performed by splitting each HisA sequence into four modules (HisA1-HisA4) following secondary structures succession in the corresponding protein from *Thermotoga maritima*, whose three-dimensional structure is available (we will refer to these four regions as the "quarters"). The alignments concerning *Methanocaldococcus jannaschii *are shown in Figure 2 (identity and similarity values are in Table 1). The degree of sequence similarity is not very high but it might be used to support an evolutionary model suggesting that the present day situation could have been reached after two gene elongation events, each one doubling the length of the ancestral gene and the number of (*β*/*α*)-modules in the product. Thus the HisA TIM-barrel would be the result of a cascade of two consecutive gene elongations (Figure 3). This model is supported by the structures shown in Figure 3 (upper left panel). We used the *T. maritima *HisA structure (1Q02) to obtain the coordinates of each atom composing the "quarters" of barrel and then performed a structural superposition using swiss PDB viewer. Lang et al., [19] compared the structure of the two HisA half-barrels and obtained a *root-mean-square *deviation (rms) ranging from 1.5 to 2.0 Å using all main chain, non hydrogen atoms. Our results concerning "quarters" structural superpositions showed for the first and the second quarters an average rms of 1.21 Å using all alpha carbons, strongly supporting the model proposed. We showed examples from these two organism, because *M. jannaschii *with several other Archaea showed the best overall degree of conservation of internal repetitions, while the choice of *T. maritima *followed the availability of HisA tridimensional structure [19].

**Table 1:** Identity and similarity values for the HisA quarters comparisons.

HisA quarters comparisons				
	*hisA1*	*hisA2*	*HisA3*	*hisA4*

*HisA1*		15.8	29	25.4
*HisA2*	42.9		20.6	32.2
*HisA3*	58.1	42.9		13
*HisA4*	42.9	58.1	41.9	

Identity (upper diagonal) and similarity percentages calculated for the alignments shown in Figure 2.

The symmetry of the TIM-barrel structure suggests to test a further ancestral duplication in which the original gene coded for a single (*β*/*α*)-module, capable of forming a homo-octamer to form a complete barrel. Although the alignments constructed from the eight single (*β*/*α*)-modules are very short, they still contain a non negligible amount of sequence and secondary structure similarities not expected from random distribution of amino acids and by visual comparison of their structure is in agreement with this hypothesis (Figure 3, lower left panel). Thereby, the ancestral forms of life might have expanded their coding abilities and their genomes by duplicating a small number of mini-genes, i.e. the "starter types". We are completely aware that the evidence of the ancient duplications involving an ancestral mini-gene encoding the "quarter" of barrel and the single (*β*/*α*)-module is based on very limited amount of sequence and structural similarities; in spite of this, in our opinion, the hypothesis mentioned above remains valid. Moreover, further studies are needed to clarify the presence of these additional gene elongation events and to integrate them into a more general picture of the evolution of the very diversified TIM-barrel family of proteins.

### The *hisNB *fusion

The eighth and sixth steps of histidine biosynthesis are catalyzed by histidinol-phosphate phosphatase (EC 3.1.3.15) (HOL-Pase) and IGP dehydratase (EC 4.2.1.19), respectively [1]. Distinct HOL-Pases have been characterized in different organisms, whereas IGP dehydratase is the same in all known histidine synthesizing organisms. In *E. coli *the two activities are coded for by a single gene, referred to as *hisNB *[11]: the N-terminal domain (HisN) is a phosphatase belonging to the DDDD family [11] and the C-terminal domain is responsible for IGP dehydratase activity. The evolutionary history of the *hisNB *gene has been recently reported by [11] who showed that *hisNB *gene fusions are present in most *γ*-proteobacteria and in the *ε*-proteobacterium *Campylobacter jejuni*; phylogenetic analysis allowed to trace the fusion event in an ancestor of the *γ*-subdivision and its later horizontal transfer to *C. jejuni*. Moreover, *hisN *is paralogous to *gmhB *(*E. coli *nomenclature), catalyzing the dephosphorylation of D-*α*-D-heptose 1,7-PP for surface Lipopolysaccharide production [20,21].

Since the former *hisNB *evolutionary model was based on a limited number of genomes, we update it including all available genomes (April, 1: 41 Archaea, 759 Bacteria and 135 Eukarya).

**Figure 4:**
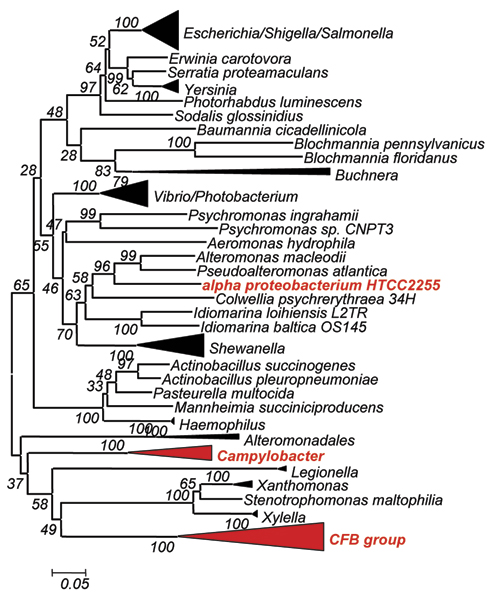
**HisNB phylogenetic analysis**. HisNB Phylogenetic tree. Organisms (groups) in red are bacteria not belonging to γ-proteobacteria and harboring the HisNB fusion.

By combining results obtained with several queries, we retrieved 131 orthologous bifunctional HisNB sequences: 104 come from *γ*-proteobacteria, 9 from *ε*-proteobacteria, 1 to a *α*-proteobacterium and 17 from the CFB group (Table 2 and Additional file 1). No archaeal and eukaryotic bifunctional sequence was retrieved although they possess genes encoding DDDD-type phosphatases, or, more generally HAD hydrolases [11]. These data confirmed the narrow phylogenetic distribution of the *hisNB *fusion, which is mostly present in *γ*-proteobacteria. However, the occurrence of a fused *hisNB *gene in other lineages enlarged its distribution raising the question of the origin of this fusion in these phyla, i.e. if it is either the outcome of convergent evolution or a horizontal gene transfer event (HGT). To discern between these two different scenarios, a phylogenetic analysis of HisNB sequences was carried out. A phylogenetic tree obtained using a representative set of HisNB sequences is reported in Figure 4, which shows that HisNB sequences from *α*- and *ε*-proteobacteria, and CFB bacteria are intermixed with *γ*-proteobacterial sequences and do not reflect the 16S rDNA phylogeny. This result strongly suggests that the *hisNB *gene has been horizontally transferred from some *γ*-proteobacteria to the other microorganisms. It is also quite possible that the transfer event might have involved the entire *his *operon or part thereof, as evidenced by phylogenetic trees of other *his *genes (e.g. Additional File 2). This statement relies on the analysis of the organization of *his *genes in the 131 genomes harboring the *hisNB *fusion, which revealed (Additional file 3) that all of them are localized within more or less compact operons.

**Table 2:** Phylogenetic distribution of *HisIE *and *HisNB *genes.

**Domain**	**Phylum**	**Class**	**# His+**	**% HisIE**	**% HisNB**
Bacteria	Acidobacteria	Acidobacteria	1	0	0
		Solibacteres	1	0	0
	Actinobacteria	Actinobacteria	36	2.8	0
	Aquificae	Aquificae	1	100	0
	Bacteroidetes	Bacteroidetes	2	100	50
		Flavobacteria	1	100	100
		Sphingobacteria	1	100	100
	Chlorobi	Chlorobia	4	0	0
	Chloroflexi	Dehalococcoidetes	2	0	0
	Cyanobacteria	Chroococcales	10	90	0
		Gloeobacteria	1	100	0
		Nostocales	2	100	0
		Oscillatoriales	1	100	0
		Prochlorales	10	100	0
	Firmicutes	Bacilli	37	48.7	0
		Clostridia	10	80	0
	Planctomycetes	Planctomycetacia	1	0	0
	Proteobacteria	*α*	52	1.9	1.85
		*β*	39	0	0
		*δ*	12	16.7	0
		*ε*	7	85.7	82
		*γ*	89	68.5	95
	Spirochaetes	Spirochaetes	4	0	0
	TD group	Deinococci	4	100	0
	Thermotogae	Thermotogae	1	100	0
Archaea	Crenarchaeota	Thermoprotei	6	0	0
	Euryarchaeota	Archaeoglobi	1	0	0
		Halobacteria	4	0	0
		Methanobacteria	2	0	0
		Methanococci	3	0	0
		Methanomicrobia	8	0	0
		Methanopyri	1	0	0
		Thermococci	2	100	0
		Thermoplasmata	1	100	0

Phylogenetic distribution analysis of the fusions HisIE and HisNB, along with the percentage of species possessing one of them (two right – most columns) on the number of histidine producing organisms (as assessed by the presence of *his *genes in their genome). Eukarya are treated separately (see HIS4 section) and never possess the *hisNB *gene fusion.

### The *hisIE *fusion

The *hisI *and *hisE *genes code for a phosphoribosyl-ATP phosphohydrolase and a phosphoribosyl-AMP cyclohydrolase that are responsible for the third and second steps in histidine biosynthesis, respectively. In *E. coli *and *S. enterica *the two genes are fused to form the last gene of the *his *operon (Figure 1).

The phylogenetic distribution of *hisIE *genes was obtained by retrieving homologous sequences using the *E. coli *HisIE amino acid sequence as a query to probe genome database. The data are reported in Table 2 and can be summarized as follows (see also Additional file 4):

1. The *hisIE *fusion is not universally distributed;

2. Bifunctional *hisIE *genes were found in all eukaryotes (see section regarding *HIS4*);

3. Most of the archaeal genomes harbor monofunctional *hisI *and *hisE *genes; the occurrence of *hisIE *in Thermococci and Thermoplasmata is very likely the outcome of a HGT event from a bacterium donor [15]. Moreover, when the *hisI *and *hisE *genes are not fused in Archaea, they do not belong to operons and are separated on the chromosome. The only exceptions are represented by *Sulfolobus *species, where the two genes are in a compact operon but separated by the *hisH *gene;

4. The *hisIE *gene fusion is present in 100% of the histidine producing organisms belonging to Aquificae, Deinococci, Bacteroidetes, Cyanobacteria, and Thermotogae. Moreover, a bifunctional *hisIE *gene was found in all *γ*-proteobacteria that branched off after the separation of Pseudomonadales from the main branch. The presence of the fusion in *ε*-proteobacteria can be explained by means of a HGT of the entire operon [13] from *γ*-proteobacteria; the same appears to be true for Bacteria belonging to the CFB group and possessing the *hisNB *gene fusion (see the corresponding paragraph). In spite of the high number of genomes sequenced (39), no *hisIE *fusion was found in *β*-proteobacteria, which represent the key-point for the compacting of *his *genes during the construction of proteobacterial *his *operon [13].

5. Firmicutes show a complex scenario: we have found fused and stand-alone genes in very closely related species of *Bacillus *(i.e. *Bacillus subtilis *possesses the gene fusion while *B. thuringiensis *and *B. anthracis *do not; in these cases *hisI *and *hisE *are contiguous and very close on the chromosome); the same is true for Clostridia. The presence of the fusion in model organisms such as *Bacillus subtilis *but not in some of the recently sequenced genomes of the same genus suggests that sequencing artifacts, probably favored by the gene organization of these two genes, might explain this situation.

6. Actinobacteria lack this gene fusion.

7. Apparently there is no correlation between the occurrence of *hisIE *fusion and *his *genes organization. However, it is interesting that during Proteobacteria evolution, we witness a progressive approaching of two initially far *hisI *and *hisE *cistrons, starting from *δ*- and *ε*-proteobacteria. The distance between them decreases in bacteria belonging to the *α*-branch; then, they partially overlap in *β*-proteobacteria, a molecular event which is coincident with the formation of a complete *his *operon, which very often includes genes apparently not involved in histidine biosynthesis. Finally, the two genes fused in the ancestor of *γ*-proteobacteria, where the compactness of *his *operon is very high [13].

Despite of the proposed HGTs, the current phylogenetic distribution of the HisIE bifunctional enzyme evokes a scenario of convergent evolution and independent gene fusions/splitting of the two cistrons in different lineages. However, phylogenetic analyses are not of great help to confirm this view (data not shown) because these proteins are very short (less than 100 residues each) and the informative sites in a multialignment of sequences coming from complete genomes are extremely reduced bringing to unreliable trees (i.e. very low bootstrap support, star topologies, data not shown). On a different perspective, the analysis of the linker region connecting the two domains might help in understanding the evolutionary history of these fusions, but we have found that they are very short, giving no information on this issue (data not shown). If the idea on convergent evolution of these gene fusions will hold future works, it might turn out that there is a strong selective pressure favoring their appearance in different lineages. Lastly, the finding that the gene order in all the bifunctional genes is always *hisI*, *hisE*, suggests that a different arrangement of the two domains should be disadvantageous for the enzymatic activity and that structural and/or functional constraints might be responsible for the extant arrangement.

### From metabolons to multifunctional enzymes: the eukaryotic HIS7 and HIS4 fusions

Two fusions involving *his *genes were disclosed in the yeast *Saccharomyces cerevisiae*: *HIS7*, corresponding to the bacterial *hisH *and *hisF *genes [12], and *HIS4*. The latter gene codes for a tetrafunctional enzyme consisting of about 800 residues and containing three regions homologous to the prokaryotic *hisI*, *hisE *and *hisD *genes, arranged in this order in the yeast gene, whose products perform the second, the third and the last two steps of histidine biosynthesis, respectively.

#### The IGP synthase coding gene: HIS7

**Figure 5:**
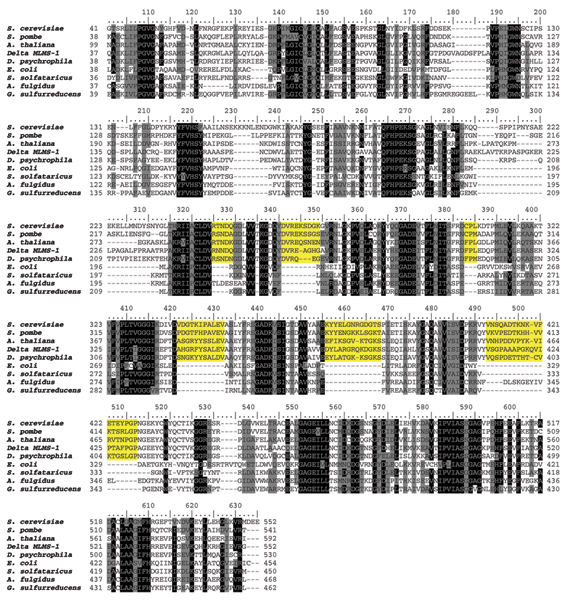
**His7 multialignment**. A multialignment of HIS7, HisHF and a set of representative concatenated HisH and HisF sequences from *E. coli*, *S. solfataricus*, *A. fulgidus *and *G. sulfurreducens*. Yellow shading represent insertions shared only by bifunctional HIS7 and HisHF proteins. Shading of the multialignment has been made with PAM250 matrix.

**Figure 6:**
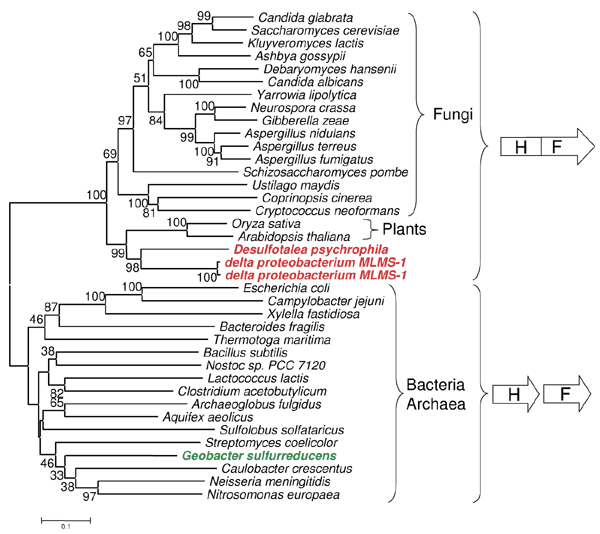
**His7 phylogenetic analysis**. Phylogenetic tree of HIS7, HisHF and concatenated HisH and HisF sequences. See Methods for details on phylogenetic tree construction.

The bifunctional enzyme IGP synthase catalyzes the fifth step of histidine biosynthesis, generating the imidazole ring of the histidine precursor, IGP. The overall reaction is the conversion of PRFAR to IGP and AICAR [2]* via *a glutamine molecule, and with no free intermediate (Figure 1). This represents the central step of the pathway, which connects histidine biosynthesis to nitrogen metabolism and the *de novo *synthesis of purines, through AICAR. The active form of the *E. coli *IGP synthase is a heterodimer of the *hisH *and *hisF *products i.e. a glutamine amidotransferase (GAT) and a cyclase, respectively [22]. The requirement for a direct interaction between GAT and the cyclase was confirmed by the discovery of the structure of the *S. cerevisiae *HIS7 gene coding for IGP synthase [23]; the analysis of the encoded enzyme demonstrated that it is constituted by two domains, an N-terminal and a C-terminal one, sharing a high degree of sequence similarity with known HisH and HisF, respectively. Previous works suggested that the eukaryotic *HIS7 *gene is the outcome of a fusion event involving two monofunctional, bacterial-like genes [12]. According to the model proposed, the eukaryotic lineage inherited two monofunctional genes, *hisH *and *hisF*, that underwent gene fusion. The alternative scenario, that is the possibility of a HGT event from a prokaryote harboring a fused *hisHF *gene to eukaryotes was excluded on the basis of the absence of the HisHF fusion in prokaryotes. However, the increasing number of sequence in databases opens the possibility to modify this model. To this purpose the *S. cerevisiae HIS7 *aminoacid sequence was used in a BLASTP search [24], allowing to retrieve 21 bifunctional sequences. Whilst most (18) come from Eukarya, three of them belonged to two δ-proteobacteria, raising the possibility that the bacterial and the eukaryotic HisHF sequences share a common ancestry, even though a phenomenon of convergent evolution could not be ruled out. Thus, all the available HisHF bifunctional sequences and a set of bacterial and archaeal concatenated HisH and HisF sequences were aligned using the program ClustalW [25]. The multialignment (partially shown in Figure 5) allowed to detect several conserved insertions that distinguish bifunctional proteins. This speaks towards a common origin of the eukaryotic and bacterial *hisHF *genes rather than a phenomenon of convergent evolution. The phylogenetic tree obtained using the above mentioned multialignment showed in Figure 6 supports this view: it can be splitted into two main clusters, one containing all the monofunctional sequences including the one coming from the *δ*-proteobacterium *Geobacter sulfurreducens*, the second one comprising all the bifunctional eukaryotic and bacterial sequences; the three bifunctional bacterial sequences clustered with Plants sequences. This body of data suggests that a bifunctional *hisHF *gene might have been transferred from Plants to some *δ*-proteobacteria.

#### A tetrafunctional gene: HIS4

The *S. cerevisiae HIS4 *gene codes for a tetrafunctional enzyme and consists of three regions sharing a high degree of sequence similarity with prokaryotic HisI, HisE, and HisD, thus the activities performed by the *HIS4 *enzyme are, from the N-terminal end, a phosphoribosyl-ATP pyrophosphohydrolase, a phosphoribosyl-AMP cyclohydrolase and the two-step histidinol dehydrogenase. The first one (HisI) uses N'-5'-phosphoribosyl-ATP (PR-ATP) to produce N'-5'-phosphoribosyl-AMP (PR-AMP), whose purine ring is subsequently opened by the second (HisE) to give 5'-ProFAR. This compound, in turn, undergoes seven additional enzymatic reactions leading to histidine, the last two of which are catalyzed by histidinol-dehydrogenase (HisD) (Figure 1) i.e. the double oxidation of histidinol to histidine, through the intermediate histidinal, concomitant to the reduction of two NAD+ molecules, with a Bi-Uni-Uni-Bi kinetic mechanism [26,27].

##### Sequence retrieval and hisI, hisE and hisD gene structure in Eukarya

The eukaryotic complete genomes database of protein sequences was probed using the HisIE and HisD domains of the *S. cerevisiae HIS4 *enzyme (residues 134–329 and 351–795, respectively). The BLASTP [24] search returned the eukaryotic sequences listed in Table 3 and revealed that the two sequences used as queries retrieved two identical sequences, with the exception of *Aspergillus nidulans *(where an additional protein with a putative HisD function was retrieved), *Schizosaccharomyces pombe *and Plants, where genes corresponding to the prokaryotic *hisIE *and *hisD *counterparts were detected.

**Table 3:** Phylogenetic distribution of *HIS4 *genes.

**Taxonomy**			**Organism**	**Strain**	**Protein GI**	**Length (aa)**	**Gene structure**			
							*	**I**	**E**	**D**
**Ascomycota**	**Pezizomycotina**	**Eurotiomycetes**	*Aspergillus nidulans*	FGSC A4	40743835	438				**X**
					40746471	867	**N**	**X**	**X**	**X**
		**Sordariomycetes**	*Gibberella zeae*	PH-1	42547615	854	**N**	**X**	**X**	**X**
			*Magnaporthe grisea*	70–15	38109852	865	**N**	**X**	**X**	**X**
			*Neurospora crassa*		32420263	870	**N**	**X**	**X**	**X**
	**S. mycotina; S. mycetales**	**Dipodascaceae mitosporic S. mycetales**	*Yarrowia lipolytica*	CLIB99	50545145	855	**N**	**X**	**X**	**X**
			*Candida albicans*		3757752	838	**N**	**X**	**X**	**X**
			*Candida glabrata*	CBS138	50285163	802	**N**	**X**	**X**	**X**
		**Saccharomycetaceae**	*Debaryomyces hansenii*	CBS767	50424339	861	**N**	**X**	**X**	**X**
			*Eremothecium gossypii*		45199222	806	**N**	**X**	**X**	**X**
			*Kluyveromyces lactis*	NRRL Y-1140	50304609	795	**N**	**X**	**X**	**X**
			*Pichia pastoris*		3203	844	**N**	**X**	**X**	**X**
			*Saccharomyces cerevisiae*		10383761	799	**N**	**X**	**X**	**X**
	**Schizosaccharomycetes**		*Schizosaccharomyces pombe*		19112678	439				**X**
					19112622	417	**N**	**X**	**X**	
**Basidiomycota**	**Hymenomycetes**	**Heterobasidiomycetes**	*Cryptococcus neoformans*		50258877	852	**N**	**X**	**X**	**X**
			*Hebeloma cylindrosporum*		31095443	843	**N**	**X**	**X**	**X**
**Viridiplantae**	**Ustilaginomycetes Brassicaceae**		*Ustilago maydis*	521	46099735	896	**N**	**X**	**X**	**X**
			*Arabidopsis thaliana*		10177677	467				**X**
					21554409	281	**S**	**X**	**X**	
			*Brassica oleracea*	var. capitata	99844	469				**X**
			*Thlaspi goesingense*		3982577	464				**X**
	**Magnoliophyta**	**Poaceae**	*Oryza sativa*	japonica	34904356	459				**X**
					34903270	202	**S**	**X**	**X**	

Summary of results obtained for HIS4 genes. Right-most columns indicate the presence of a given domain in the corresponding protein; column marked with an asterisk corresponds to the N – terminal region found in Fungi (N), of unknown function, and Plants (S), a signal peptide for chloroplast localization.

**Figure 7:**
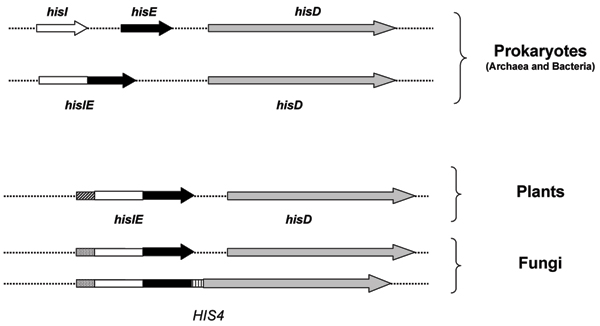
***HIS4*, *hisIE *and *hisD *genes**. Schematic representation of the archaeal, bacterial and eukaryotic genes coding for phosphoribosyl-ATP pyrophosphohydrolase (*hisI*), phosphoribosyl-AMP cyclohydrolase (*hisE*) and histidinol dehydrogenase (*hisD*). The HisI and HisE proteins are coded by a bifunctional gene or two separate cistrons in both Archaea and Bacteria and by a bifunctional gene in all Eukarya. Homologus regions are represented by the same hatching.

A multialignment of all the retrieved sequences with a representative set of archaeal and bacterial HisIE and HisD sequences (Additional file 5) revealed that the *HIS4*-like genes can be subdivided into four portions (Figure 7): i) an N-terminal region of variable length, followed by ii) the *hisIE *moiety, which in turn precedes iii) a linker region of variable sequence and length connecting the *hisIE *region to the last domain, iv) the *hisD *region. Plants sequences have an N-terminal region which is unrelated to any histidine biosynthetic enzymes and that might represent a signal sequence for chloroplast localization, an idea which is in agreement with a (at least partial) compartmentalization of histidine biosynthesis into these organelles, as previously suggested [28]. A similar search performed on prokaryotic databases did not allow retrieving any HIS4-like protein.

##### Analysis of the HIS4 N-terminal region

The fungal HIS4 sequences have an N-terminal domain (whose length ranges from 160 to 220 residues) that is much longer than that found in HisIE from Plants; moreover, we detected no significant homology neither with the signal sequence found in Plants nor with any other signal sequence of Fungi or other organisms (data not shown). This sequence has no known conserved domains, as appeared by the absence of hits in the Conserved Domain Database (data not shown). Moreover, a psi-blast search did not permit to obtain any statistically significant hit, if we exclude other HIS4 proteins (data not shown). However, both the presence of the corresponding sequence in mRNAs (see GenBank entry NM_212387.1 from *Ashbya gossypii*) and the molecular weight of the isolated *S. cerevisiae *HIS4 enzyme (95000 ± 500 Da) [29] speak toward the presence of this N-terminal sequence in the "final polypeptide". A structural rather than a catalytic role of this region can be suggested on the basis of alignment of the isolated N-terminal regions of the fungal HIS4 enzymes, which revealed that the degree of sequence similarity between them is quite low and significantly less than that shared by catalytic domains (Additional file 6 and 7).

##### Phylogenetic analyses

**Figure 8:**
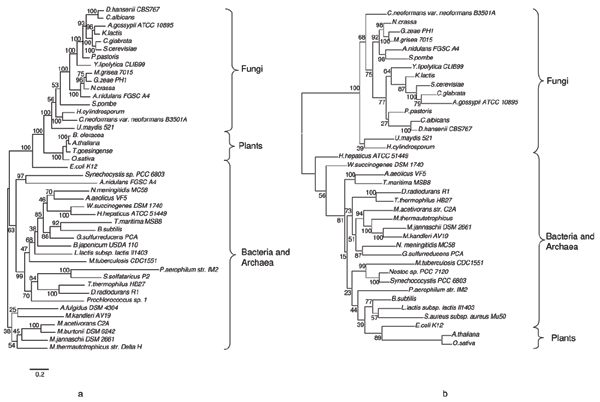
**HisD and HisIE phylogenetic analysis**. Phylogenetic tree obtained using a multialignment of HisD (a) and HisIE (b) proteins and domains from HIS4 proteins and MrBayes program. Values above nodes are posterior probabilities*100 (for details on phylogenetic tree construction see Methods). See text for details.

A phylogenetic analysis was performed to check whether HisIE and HisD proteins/domains listed in Table 3 experienced the same evolutionary history and whether the phylogenetic trees were congruent with the phylogeny of Eukarya based on other molecular markers, such as 18S rDNA. According to the eukaryotic phylogeny based on 18S rDNA [30] Viridiplantae branched off from the major eukaryotic line earlier than Fungi, that represent a sister group of Metazoa. Moreover, within Fungi, Basidiomycota appear to branch off earlier than Ascomycota; the latter are rooted by Archiascomycotina, including Schizosaccharomycetes. However, discordant phylogenies have been obtained using different sequences and this might be mainly due to HGTs or to the transfer of organellar genes to the nucleus [31,32]. The analysis of the HisD phylogenetic tree obtained using all the available eukaryotic sequences with their prokaryotic counterparts revealed that it is consistent with the eukaryotic species phylogeny and supported by very high bootstrap values (Figure 8a), suggesting a vertical inheritance for *hisD *in Fungi and Plants. The phylogenetic analyses also revealed that the second *Aspergillus nidulans hisD *gene has been acquired from a prokaryote, probably a Cyanobacterium. The interpretation of the phylogenetic tree obtained using the HisIE sequences is quite different (Figure 8b): two distinct clusters can be recognized, the first one comprising sequences from Fungi, and the second one including prokaryotic and Plants sequences. HisIE from Fungi now is separated from Plants, and Eukarya are not monophyletic. A possible explanation is that *hisIE *genes of Fungi and Plants have not been vertically inherited from a common ancestor; however HisIE proteins often gave rise to 'strange' phylogenetic trees for their short length.

Concerning the splitting of the two moieties (HisIE and HisD) in *S. pombe*, the presence of the N-terminal unknown sequence in the *hisIE *gene product (Table 3) and the phylogenetic trees reported in Figures 7 suggest a *HIS4 *gene fission event rather than its primary absence. Moreover, the fission yeast belongs to the Ascomycota, all of which possess the canonical *S. cerevisiae*-like HIS4 enzyme, and the same (Table 3) is true for species which branch off earlier from the fungal lineage (as the Basidiomycota *Cryptococcus neoformans *and other).

##### An evolutionary model for the origin and evolution of *HIS4 *gene

A possible evolutionary model for the origin and evolution of *HIS4 *predicts that (at least) one copy of *hisD *was donated from prokaryotes to the ancestor of Eukarya and this sequence was vertically inherited by Fungi and Plants. Concerning *hisIE*, it is quite possible that the ancestor of eukaryotes received a bifunctional *hisIE *gene from prokaryotes rather than two monofunctional genes that then underwent a gene fusion. These ideas are consistent with both the structure of *hisIE *genes in known Eukarya and with the phylogenetic trees shown in Figure 8. However, the ancestor of Fungi and Plants might have received the HisIE gene from different prokaryotes, as shown by the HisIE phylogenetic tree (Additional file 8). After the divergence of Fungi from Plants the fusion between the two bifunctional genes (*hisIE *and *hisD*) occurred in Fungi leading to the extant *HIS4 *tetrafunctional gene, which was maintained during the evolution with the exception of *S. pombe*, where it was split.

## Conclusion

In this paper we have analyzed the fusions involving histidine biosynthetic genes. At least eight out of ten *his *genes, i.e., *hisA*, *B*, *D*, *E*, *F*, *H*, *I*, and *N *underwent different fusion events strongly supporting a major role of this mechanism in both the assembly and evolution of histidine biosynthesis. Each of the five *his *fusions detected so far, i.e. *hisA/hisF*, *hisIE*, *hisHF *(*HIS7*), *hisNB*, and *hisIED *(*HIS4*) has been analyzed for: i) gene structure, ii) phylogenetic distribution, iii) timing of appearance, iv) horizontal gene transfer, v) correlation with gene organization, and vi) biological significance. Our results might be summarized as follows:

1. The only "universal" gene fusion concerns *hisA *and *hisF *genes, which are the outcome of a cascade of (at least) two gene elongation events followed by a paralogous gene duplication. The structure of *hisA *and *hisF*, that is the presence of two paralogous modules half the size of the entire gene, is the same in all histidine-synthesizing organisms and no correlation with *his *genes organization exists, in the sense that the two genes maintain the same structure independently from *his *gene organization (complete or partial clustering or scattering). It is also interesting that the traces of the two elongation events are detectable at the primary sequence level in only a few species, mainly Archaea.

This suggests that the two elongation events are very ancient i.e. they date before LUCA. The analysis of the sequence and structure of HisA and HisF depicts a likely scenario for divergent evolution of (at least) some of the proteins belonging to the TIM-barrel family; interestingly HisA is the only one maintaining an almost perfect subdivision in two modules half the size of the entire gene and sharing a high degree of sequence similarity. In other TIM-barrels, such as HisF and TrpF, the common origin of the two halves has been obscured by point mutations and/or larger rearrangements due to functional and/or structural constraints. Therefore it is possible that HisA might resembles the ancestral TIM-barrel enzyme. By structural comparisons of fragments of the *T. maritima *HisA protein we obtained indications on the paralogy between quarters of the barrel (each correspondingto a (*β*/*α*)_2 _module).

2. No fusion involving *his *genes has been disclosed in Archaea, with the exception of *hisIE *in some Euryarchaeota. However, the *hisIE *bifunctional genes are very likely not native of those Archaea, but are the outcome of a HGT event involving an entire bacterial *his *operon [15].

3. The fusion between *hisI *and *hisE *occurred more than once in Bacteria, speaking towards a phenomenon of convergent evolution; in many cases it has been preceded by the progressive approaching and overlapping of the genes (e.g. in proteobacteria). Sometimes, the fusion was concomitant with the formation of compact operons. Moreover, this gene might have been horizontally transferred.

4. The *hisNB *fusion is a relatively recent evolutionary event that happened in the *ε*-branch of proteobacteria. This fusion was parallel to the introgression of *hisN *into an already formed and more or less compact *his *operon. Once occurred, the fusion was fixed and transferred to other proteobacteria and/or CFB group along with the entire operon or part thereof.

5. The fusions involving *hisH *and *hisF*, *hisIE *and *hisD *occurred in the eukaryotic lineage. Whilst the fusion leading to the tetrafunctional gene *HIS4 *is peculiar of eukaryotes, a *hisHF *fusion was found also in two bacteria, probably as a result of a HGT from an eukaryote.

**Figure 9:**
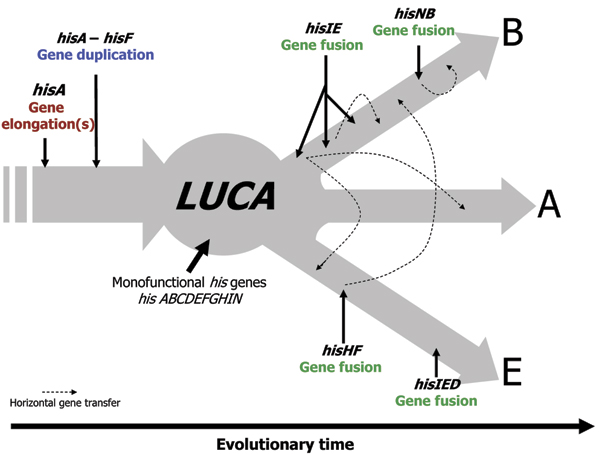
**A global view of *his *gene fusions appearance**. Schematical representation of *his *gene fusion appearance and horizontal transfer. Abbreviations used: A, B, E correspond to Archaea, Bacteria and Eukarya, respectively. LUCA stands for the Last Universal Common Ancestor.

The more or less narrow phylogenetic distribution of these fusions raises the question of the structure of *his *genes in the LUCA. On the basis of the available data, we suggest (Figure 9) that LUCA possessed all monofunctional histidine biosynthetic genes.

The whole body of data reported above suggests that the fusion(s) of histidine biosynthetic genes has been driven by different selective pressures. In the case of the elongation events leading to the extant *hisA *and *hisF*, a structural/functional significance can be invoked. Indeed, the elongation events were very likely positively selected in order to optimize the structure and the function of the ancestral TIM-barrel.

The fusion of HOL-P phosphatase and IGP dehydratase might have been selected to ensure a fixed ratio of gene products that function in the same biochemical pathway. Concerning the *hisHF *(*HIS7*) fusion, its biological significance is clear; whilst in prokaryotes the two proteins encoded by *hisH *and *hisF *must interact in a 1:1 ratio to give the active form of IGP synthase, in the eukaryotic bifunctional protein, the two entities are fused allowing their immediate interaction and the substrate tunnelling. A similar "substrate channeling" and/or "fixed ratio of gene products" might be invoked for the fusion involving the prokaryotic *hisIE *genes, which code for enzymes performing consecutive steps of histidine biosynthesis.

Independently from their case-by-case biological significance, such associations might be responsible for a more specific commitment of intermediates in a given pathway by means of the spatial co-localization of enzymes. Operons might allow Bacteria to reach the same target: the translation of polycistronic mRNAs favors protein-protein interactions or the spatial segregation of a pathway. Indeed, genes coding for interacting proteins are often organized in operons [33]; in this context, it has been suggested that the bacterial IGP synthase might be part of a complex metabolon whose entities are encoded by the four genes *hisBHAF*, constituting the so-called "core" of histidine biosynthesis [9,12]. Data presented here might suggest that the polypeptides coded for by *hisI*, *hisE*, and *hisD *are part of another metabolon.

This scenario can clarify the biological significance and the evolutionary advantages of the fusion leading to the HIS7 and HIS4 proteins and their prokaryotic counterparts. Indeed, the cytoplasm of a prokaryotic or eukaryotic cell represents an extremely complex and crowded environment, where al lot of macromolecular structures might represent an important barrier to the free diffusion of (even small) polypeptides; it is plausible that the stochastic movement of proteins that have to interact in the bacterial cytoplasm is a rate-limiting step for a pathway. It has been observed that the diffusion coefficient of many molecules in prokaryotic and eukaryotic cells is less than in water [34]. Accordingly, the intracellular concentration of proteins in *E. coli *cells has been measured to range between 300 and 400 mg/ml ([35] and references therein) revealing the bacterial cell interior to be a very crowded environment. The greater the volume and complexity of the cell the greater is the obstacle to the free diffusion of intermediates or signal molecules inside the cell. The problems related to the crowding of the intracellular milieu have been proposed to be greater for eukaryotic cells, not only for the distances an intermediate have to override to reach a given catalytic site, but especially for the presence of a number of physical obstacles, as the cytoskeleton, multi-enzymatic complexes and organelles. One of the major effects of the crowding is the reduced mobility of molecules, an effect directly related to the properties and the translational ray of a molecule. Moreover many of the intermediates of metabolic pathways can be sequestered by aspecific binding to intracellular structures or be consumed by unwanted catalytic activities, reducing the overall rate of production of the end-product and augmenting its average energetic cost. If a similar view is correct, and the diffusion problem is rate limiting for at least some of the metabolic pathways performed by the cell, then the substrate channeling can permit to bypass the problem and the loss of intermediates by collateral pathways, which might result in a waste of energy. In eukaryotic cells, where the operons are absent and whose inside is more complex than that of prokaryotes, this obstacle might be bypassed by the fusion of functional domains that permits an immediately active product after the translation of a single mRNA.

## Methods

### Sequence retrieval

Nucleotide and amino acid sequences were obtained from the NCBI complete genomes database. The BLASTp option of the BLAST program [24] was used to retrieve His proteins used in this work.

### Alignment and phylogenetic analyses

The ClustalW program [25] with standard parameters was used for pairwise and multiple amino acid sequences alignments, followed by careful visual inspection. Phylogenetic trees were obtained by using the software Mega3, the Neighbor-Joining method [39] with 1000 bootstrap replicates and the JTT [38] evolutionary model. We obtained different topologies for the tree corresponding to HIS4 domains and we compared them with those obtained with bayesian inference, i.e. MrBayes [36]. We defined the following parameters (not cited if default settings were used): evolutionary models of amino acid sequences were the WAG [37] and JTT [38] with character frequencies estimated from dataset (-F); topologies obtained were identical for the two model; we report the shortest trees (in both cases corresponding to the one obtained with WAG); we used heterogeneous rates among sites, distributed as a Gamma distribution with shape parameter free of variate from 0 to 50 (average obtained for these datasets 0.85); MCMC settings were as follows: 2,500,000 and 1,500,000 generations, respectively for HisIE and HisD domains, and five chains. No starting trees were used, with the idea in mind that convergence to very similar values of the five chains would have been more significant than starting each chain from the same tree. Trees were sampled every 250 generations, for a total set of 10,000 and 6,000 trees. Burnin was used to exclude from following analysis those trees which were sampled before convergence of the chains; this was assessed case-by-case by calculating average, standard deviation and variance. Convergence was reached after about 50,000 generations (200 trees discarded). The resulting datasets were used to obtain the shown trees with the allcompat option.

### Structural alignments

Structural alignments and Root-means-squares calculations were performed using Swiss pdb viewer [40]. We isolated modules corresponding to (*β*/*α*)_2 _and (*β*/*α*) accordingly to the analysis performed by [19] showing secondary elements belonging and not belonging to the barrel structure. These modules were then used as independent molecules and structurally aligned by using the 'magic fit' option.

## Competing interests

The authors declare that they have no competing interests.

## Authors' contributions

All authors contributed equally to the work and manuscript preparation.

## Supplementary Material

Additional file 1 **Phylogenetic distribution of *hisNB *genes**. Histogram showing the percentage of organisms possessing a *hisNB *gene for taxonomic groups represented in NCBI genomes database and taking into account only histidine producing organisms. Click here for file

Additional file 2 **HisD phylogenetic tree of organisms possessing *hisNB***. A NJ phylogenetic tree (evolutionary model: Dayhoff, 500 bootstrap replicates) obtained from a HisD multialignment. The topology is congruent with those obtained with other His proteins; it illustrates that *hisNB *has been probably transferred together with a complete histidine biosynthetic operon. See also Additional File 3 concerning gene organization. Red: *ε*-proteobacteria possessing (upper group) and not possessing (bottom group) the *hisNB *gene fusion; Green: CFB group bacteria possessing (upper group) and not possessing (bottom group) the *hisNB *gene fusion. Click here for file

Additional file 3 **HisNB and gene organization**. Several features concerning HisNB proteins from Bacteria belonging to species not available at the time of our previous analysis concerning *hisNB *genes [11]. Click here for file

Additional file 4 **Phylogenetic distribution of *hisIE *genes**. Histogram showing the percentage of organisms possessing a *hisIE *gene for taxonomic groups represented in NCBI genomes database and taking into account only histidine producing organisms. Click here for file

Additional file 5 **HIS4 multialignments with concatenated prokaryotic sequences**. A multialignment of fungal HIS4 sequences and the corresponding proteins from Plants and from a selected number of Prokaryotes. Click here for file

Additional file 6 **Entropy plot of a multialignment of HIS4 sequences**. Entropy plot of the multialignment of HIS4 proteins. The regions of the protein are also indicated showing their different degree of conservation. Entropy was calculated with the following formula: *H*(*I*) = S [*f*(*b*, *I*) * *ln*(*f*(*b*, *I*))], where b is a residue found in in column I and f(b, I) its frequency in I and the summation extends over all residues in column I. Click here for file

Additional file 7 **Pairwise identity values within HIS4 domains**. Identity values for the pairwise comparison of the different domains composing HIS4 proteins (the standard deviation is also shown). Click here for file

Additional file 8 Identifiers of sequences used in this work. Click here for file
