# Supplementary figures and images for: The role of gene fusions in the evolution of metabolic pathways: the histidine biosynthesis case
Source: BMC Evol Biol. 2007 Aug 16;7(Suppl 2):S4. doi: 10.1186/1471-2148-7-S2-S4 (PMC1963479; doi:10.1186/1471-2148-7-S2-S4)

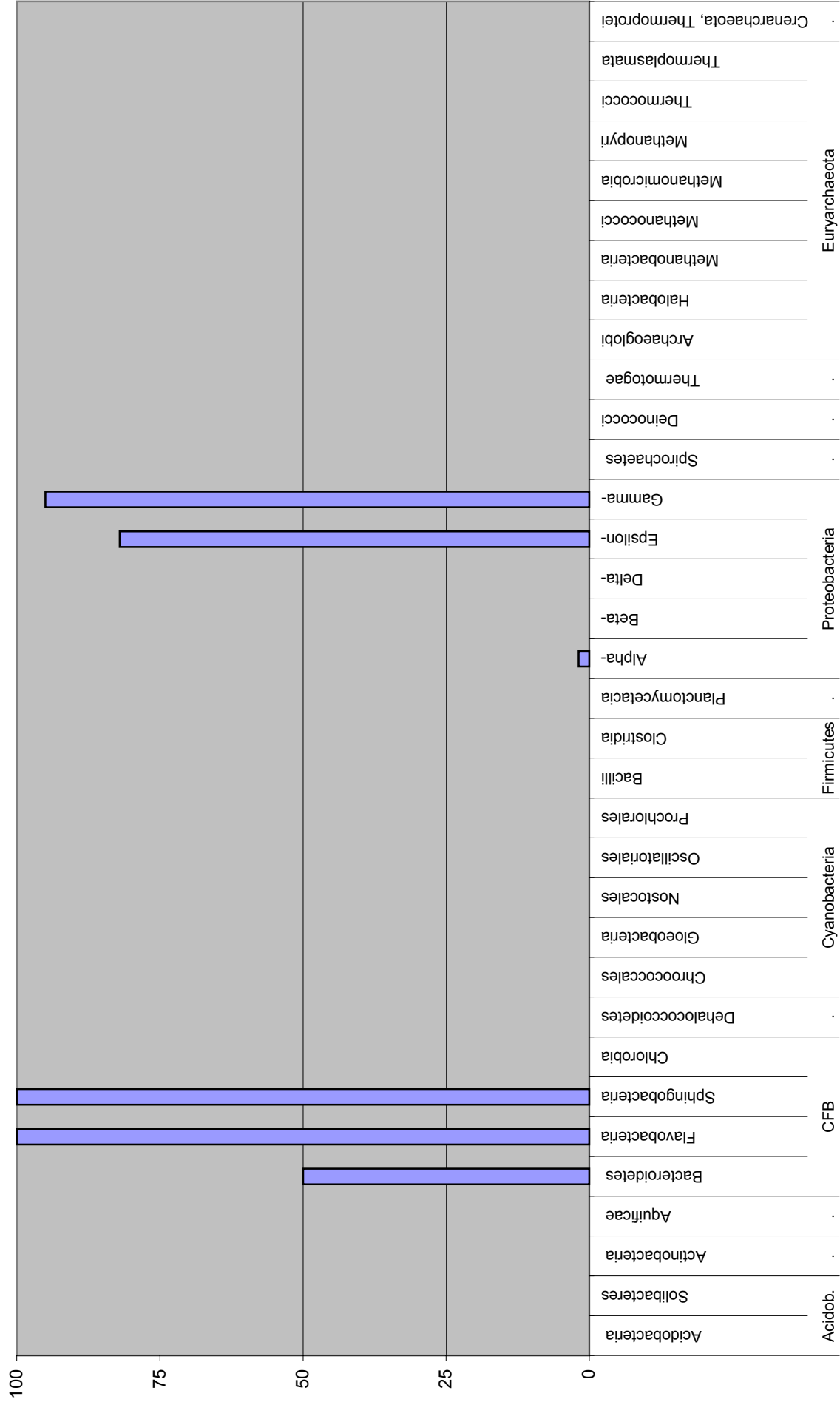

Supplement: Additional file 1 — Phylogenetic distribution of hisNB genes. Histogram showing the percentage of organisms possessing a hisNB gene for taxonomic groups represented in NCBI genomes database and taking into account only histidine producing organisms. [file 1471-2148-7-S2-S4-S1.pdf]

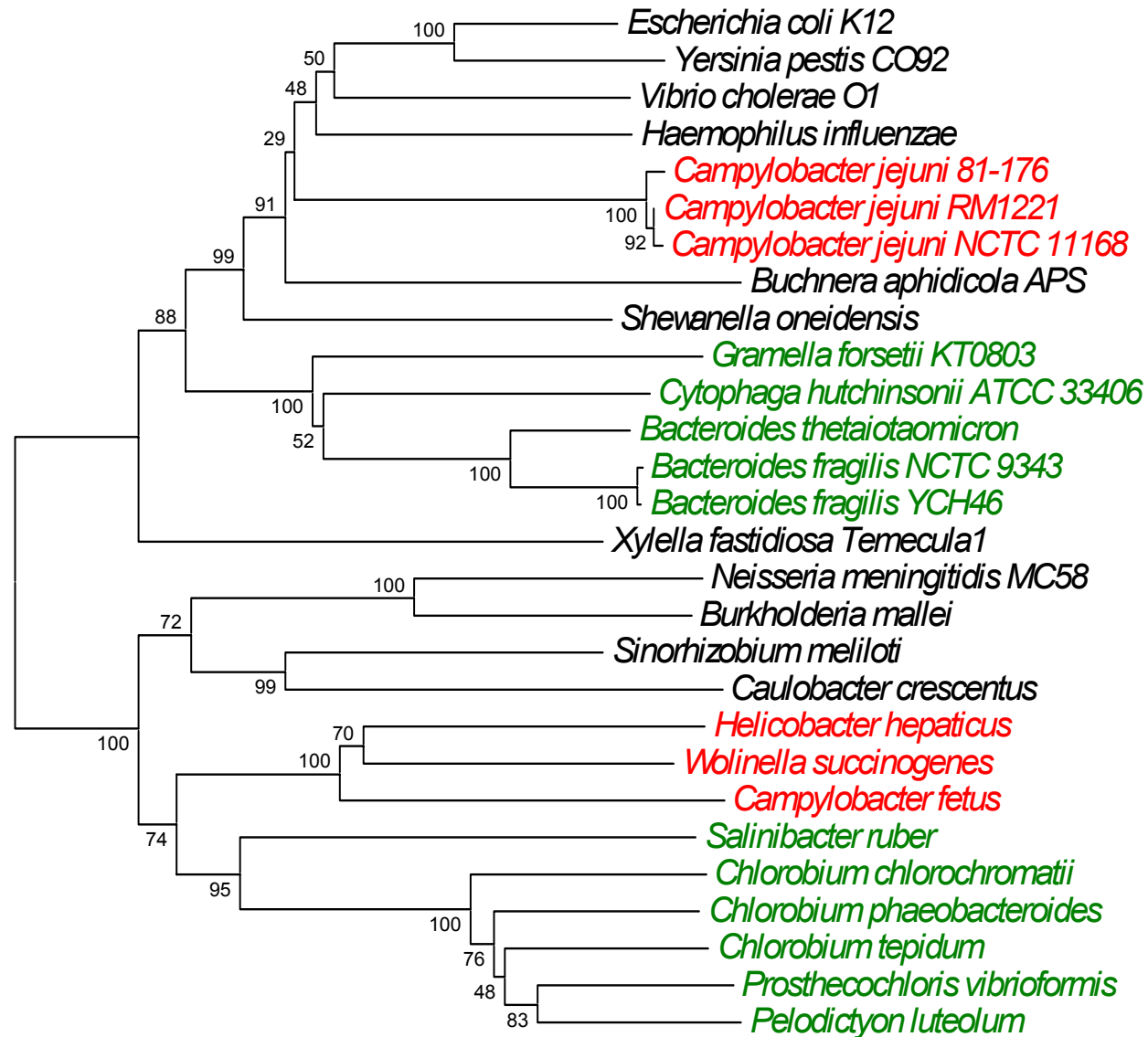

Supplement: Additional file 2 — HisD phylogenetic tree of organisms possessing hisNB. A NJ phylogenetic tree (evolutionary model: Dayhoff, 500 bootstrap replicates) obtained from a HisD multialignment. The topology is congruent with those obtained with other His proteins; it illustrates that hisNB has been probably transferred together with a complete histidine biosynthetic operon. See also Additional File 3 concerning gene organization. Red: ε-proteobacteria possessing (upper group) and not possessing (bottom group) the hisNB gene fusion; Green: CFB group bacteria possessing (upper group) and not possessing (bottom group) the hisNB gene fusion. [file 1471-2148-7-S2-S4-S2.pdf]

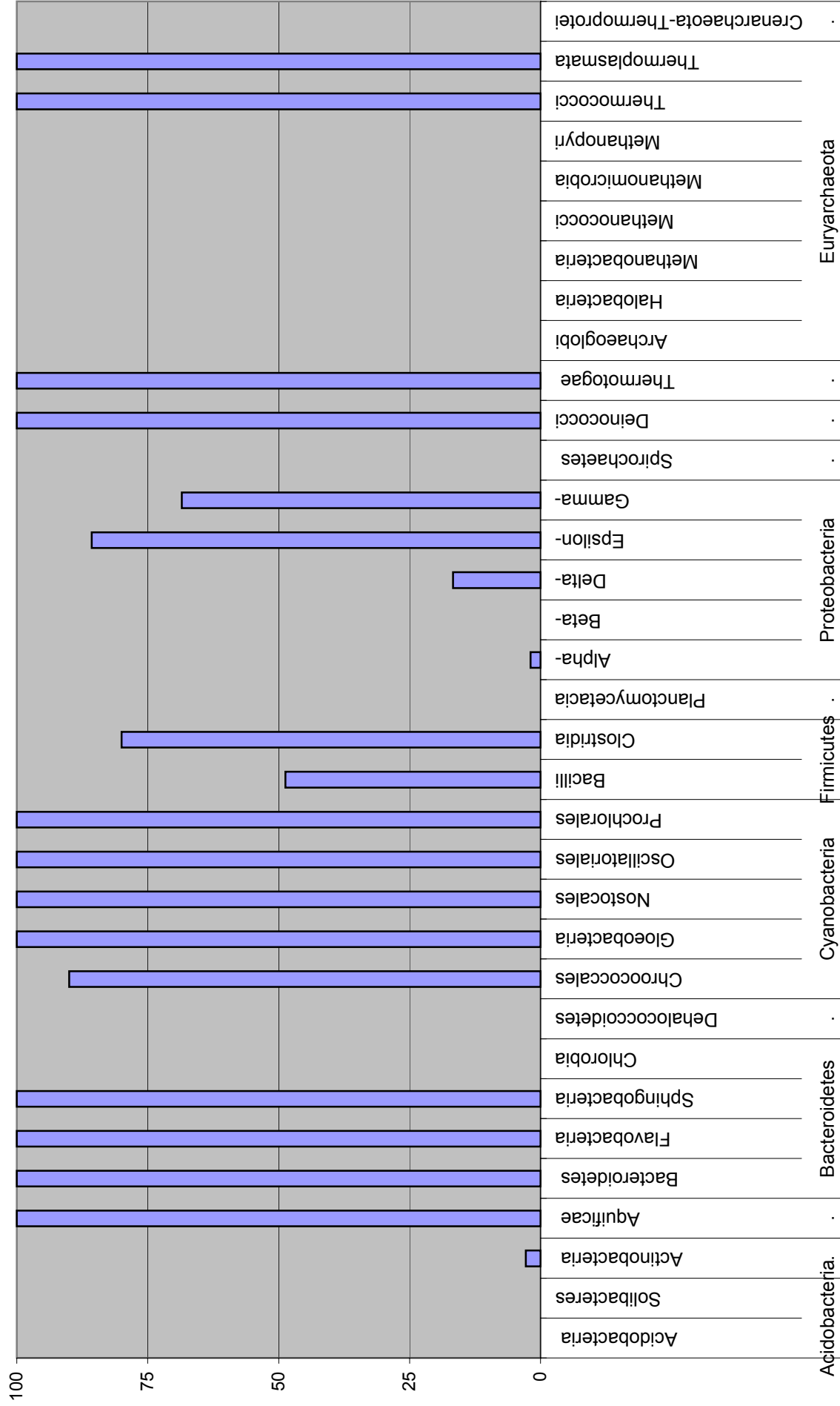

Supplement: Additional file 4 — Phylogenetic distribution of hisIE genes. Histogram showing the percentage of organisms possessing a hisIE gene for taxonomic groups represented in NCBI genomes database and taking into account only histidine producing organisms. [file 1471-2148-7-S2-S4-S4.pdf]

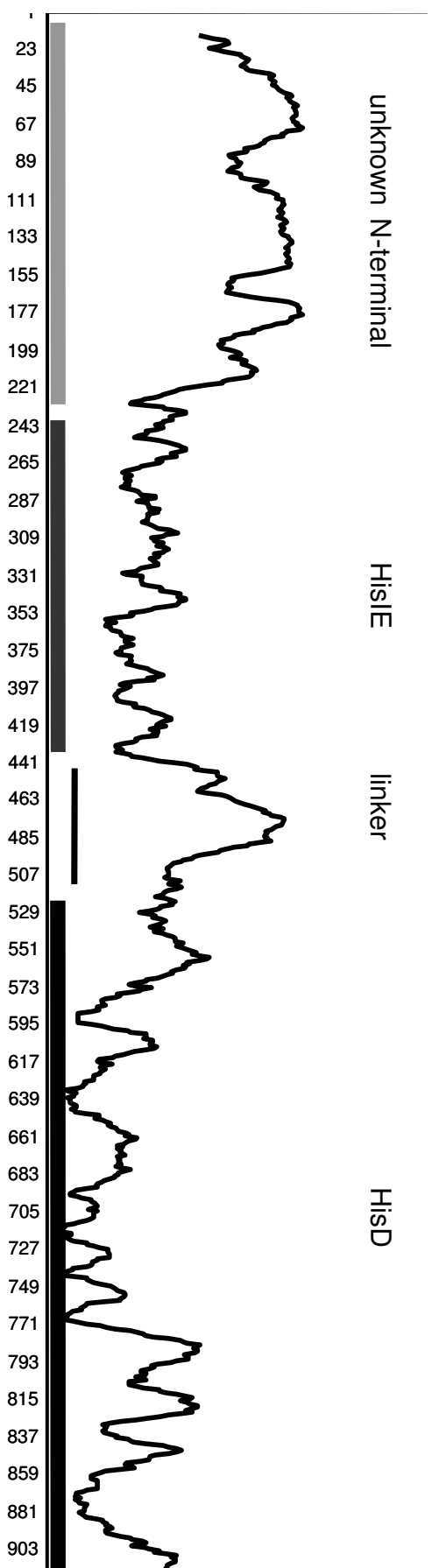

Supplement: Additional file 6 — Entropy plot of a multialignment of HIS4 sequences. Entropy plot of the multialignment of HIS4 proteins. The regions of the protein are also indicated showing their different degree of conservation. Entropy was calculated with the following formula: H(I) = S [f(b, I) * ln(f(b, I))], where b is a residue found in in column I and f(b, I) its frequency in I and the summation extends over all residues in column I. [file 1471-2148-7-S2-S4-S6.pdf]
