# Supplementary material for: The role of gene fusions in the evolution of metabolic pathways: the histidine biosynthesis case
Source: BMC Evol Biol. 2007 Aug 16;7(Suppl 2):S4. doi: 10.1186/1471-2148-7-S2-S4 (PMC1963479; doi:10.1186/1471-2148-7-S2-S4)
Supplement: Additional file 3 — HisNB and gene organization. Several features concerning HisNB proteins from Bacteria belonging to species not available at the time of our previous analysis concerning hisNB genes [11]. [file 1471-2148-7-S2-S4-S3.pdf]

Organism	Length	GI	Taxonomy	Gene organization§	Genome Status
alpha proteobacterium HTCC2255	357	114773289	a	G*XG**DC[NB]HAF[IE]---BHAFI---ZG***---additional copies (scattered)	unfinished
Bacteroides thetaiotaomicron VPI-5482	374	29345613	CFB	GDC[NB]---HAF[IE]	finished
Bacteroides fragilis YCH46	374	53714474	CFB	GDC[NB]---HAF[IE]	finished
Cytophaga hutchinsonii ATCC 33406	372	110637675	CFB	GD---C[NB]HAF---I	finished
Gramella forsetii KT0803	379	120436112	CFB	GDC[NB]HAF[IE]	finished
Croceibacter atlanticus HTCC2559	377	83858108	CFB	GDCX[NB]HAF[IE]	unfinished
Cellulophaga sp. MED134	378	86130227	CFB	GDC[NB]HA---??	unfinished
Tenacibaculum sp. MED152	379	86133445	CFB	G1G2C[NB]HAF[IE]	unfinished
Flavobacterium sp. MED217	385	86143306	CFB	GDC[NB]HAXF[IE]	unfinished
Flavobacteriales bacterium HTCC2170	377	88712457	CFB	GDC[NB]HXAFXX[IE]	unfinished
Polaribacter irgensii 23-P	378	88802980	CFB	GDC[NB]HAFX[IE]	unfinished
Robiginitalea biformata HTCC2501	388	88805687	CFB	GDC[NB]HAXF[IE]	unfinished
Flavobacteria bacterium BBFL7	369	89891713	CFB	GDC[NB]HAXF[IE]	unfinished
Flavobacterium johnsoniae UW101	378	90592003	CFB	GDC[NB]HAF[IE]	unfinished
Psychroflexus torquis ATCC 700755	377	91217706	CFB	GDC[NB]HAF[IE]	unfinished
Microscilla marina ATCC 23134	373	124006022	CFB	GDC[NB]---HA--I--E	unfinished
Algoriphagus sp. PR1	365	126645652	CFB	GDC[NB]---HAF[IE]	unfinished
Flavobacteria bacterium BAL38	379	126664303	CFB	GD1---D2C1C2[NB]HAF[IE]	unfinished
Campylobacter lari RM2100	355	57241587	e	GD[NB]HAF[IE]	unfinished
Campylobacter upsaliensis RM3195	352	57242505	e	GD[NB]XXHAXFX[IE]	unfinished
Campylobacter coli RM2228	353	57504889	e	GD[NB]HAF[IE]	unfinished
Photorhabdus luminescens subsp. laumondii TTO1	355	37525515	g	GDC[NB]HAF[IE]	finished
Yersinia enterocolitica subsp. enterocolitica 8081	355	123442984	g	GDC[NB]HAF[IE]	finished
Shewanella baltica OS155	363	126174641	g	GDC[NB]HAF[IE]	finished
Shewanella loihica PV-4	356	127513128	g	GDC[NB]HAF[IE]	finished
Erwinia carotovora subsp. atroseptica SCRI1043	355	50121509	g	GDC[NB]HAF[IE]	finished
Yersinia pseudotuberculosis IP 32953	355	51595896	g	GDC[NB]HAF[IE]	finished
Mannheimia succiniciproducens MBEL55E	365	52425945	g	GDC[NB]XXXXHXAF[IE]	finished
Legionella pneumophila subsp. pneumophila str. Phila	352	52841430	g	GDC[NB]HAF[IE]	finished
Legionella pneumophila str. Paris	352	54297154	g	GDC[NB]HAF[IE]	finished
Legionella pneumophila str. Lens	352	54294141	g	GDC[NB]HAF[IE]	finished
Photobacterium profundum SS9	361	54308282	g	GDC[NB]HAF[IE]	finished
Idiomarina loihiensis L2TR	358	56460937	g	GDC[NB]HAF[IE]	finished
Xanthomonas oryzae pv. oryzae KACC10331	375	58581881	g	GDC[NB]HAF[IE]	finished
Vibrio fischeri ES114	357	59711622	g	GDC[NB]HAF[IE]	finished
Colwellia psychrerythraea 34H	361	71282446	g	GDC[NB]HAF[IE]	finished
Candidatus Blochmannia pennsylvanicus str. BPEN	356	71892239	g	GDC[NB]HAF[IE]	finished
Shigella sonnei Ss046	356	74312565	g	GDC[NB]HAF[IE]	finished
Pseudoalteromonas haloplanktis TAC125	353	77362400	g	GDC[NB]HAXXXXXF[IE]	finished
Shigella dysenteriae Sd197	356	82777446	g	GDC[NB]HAF[IE]	finished
Shigella boydii Sb227	355	82543399	g	GDC[NB]HAF[IE]	finished
Sodalis glossinidius str. 'morsitans'	355	85059105	g	GDC[NB]HAF[IE]	finished
Shewanella denitrificans OS217	355	91792971	g	GDC[NB]HAF[IE]	finished
Baumannia cicadellinicola str. Hc (Homalodisca coagu	356	94676488	g	GDC[NB]HAF[IE]	finished
Pseudoalteromonas atlantica T6c	358	109899190	g	GDC[NB]HAFX[IE]	finished
Shewanella sp. MR-4	363	113970135	g	GDC[NB]HAF[IE]	finished
Shewanella sp. MR-7	363	114047675	g	GDC[NB]HAF[IE]	finished
Shewanella frigidimarina NCIMB 400	355	114562892	g	GDC[NB]HAF[IE]	finished
Aeromonas hydrophila subsp. hydrophila ATCC 7966	383	117618028	g	GDC[NB]HAXXXF[IE]	finished
Shewanella sp. ANA-3	363	117920296	g	GDC[NB]HAF[IE]	finished
Shewanella amazonensis SB2B	355	119775079	g	GDC[NB]HAF[IE]	finished
Psychromonas ingrahamii 37	356	119945366	g	GDC[NB]HAXXXXF[IE]	finished
Shewanella sp. W3-18-1	363	120598644	g	GDC[NB]HAF[IE]	finished
Actinobacillus pleuropneumoniae serovar 1 str. 4074	363	53729148	g	GDCX[NB]XHAF[IE]	unfinished
Shigella boydii BS512	355	75177149	g	GDC[NB]HAF[IE]	unfinished
Actinobacillus succinogenes 130Z	364	75429723	g	GDCX[NB]XH---A---F[IE]	unfinished
Shewanella putrefaciens CN-32	363	77815195	g	GDC[NB]HAF[IE]	unfinished
Yersinia bercovieri ATCC 43970	355	77959284	g	GDC[NB]HAF[IE]	unfinished
Yersinia mollaretii ATCC 43969	355	77962462	g	GDC[NB]HAF[IE]	unfinished
Yersinia frederiksenii ATCC 33641	355	77975877	g	GDC[NB]HAF[IE]	unfinished
Yersinia intermedia ATCC 29909	355	77978866	g	GDC[NB]HAF[IE]	unfinished
Shigella dysenteriae 1012	355	83571264	g	GDC[NB]HAF[IE]	unfinished
Vibrio splendidus 12B01	362	84391744	g	GDC[NB]HAF[IE]	unfinished
Idiomarina baltica OS145	358	85712681	g	GDC[NB]HAF[IE]	unfinished
Vibrio sp. MED222	362	86147092	g	GDC[NB]HAF[IE]	unfinished
Alteromonas macleodii 'Deep ecotype'	356	88794032	g	GDC[NB]HAF[IE]	unfinished
Pseudoalteromonas tunicata D2	353	88857348	g	GDC[NB]HAFX[IE]	unfinished
Photobacterium sp. SKA34	356	89075755	g	GDC[NB]HAF[IE]	unfinished
Psychromonas sp. CNPT3	356	90407647	g	GDC[NB]HAF[IE]	unfinished
Photobacterium profundum 3TCK	361	90411636	g	GDC[NB]HAF[IE]	unfinished
Vibrio angustum S14	356	90580090	g	GDC[NB]HAF[IE]	unfinished
Vibrio alginolyticus 12G01	357	91226028	g	GDC[NB]HAF[IE]	unfinished
Shewanella baltica OS195	363	113950503	g	GDC[NB]HAF[IE]	unfinished
Vibrio sp. Ex25	357	116183729	g	GDC[NB]HAF[IE]	unfinished
Yersinia pseudotuberculosis IP 31758	355	117673997	g	GDC[NB]HAF[IE]	unfinished
Serratia proteamaculans 568	355	118067988	g	GDC[NB]HAF[IE]	unfinished
Shewanella woodyi ATCC 51908	359	118074420	g	GDC[NB]HAXXXXF[IE]	unfinished
Enterobacter sp. 638	355	118739614	g	GDC[NB]HAF[IE]	unfinished
Shewanella pealeana ATCC 700345	364	118757732	g	GDC[NB]HAF[IE]	unfinished
Alteromonadales bacterium TW-7	353	119470924	g	GDC[NB]HAF[IE]	unfinished
Stenotrophomonas maltophilia R551-3	357	119879014	g	GDC[NB]HAF[IE]	unfinished

§ --- indicates a large (>10kb) separation between genes or gene clusters, X indicates a non his genes
** fragment? Only 167 residues vs average HisG >200*
*** long or E. coli type, it is autonomously feedback inhibited*
**** short type (requires HisZ for feedback inhibition and proper functioning)*
