# Supplementary material for: The role of gene fusions in the evolution of metabolic pathways: the histidine biosynthesis case
Source: BMC Evol Biol. 2007 Aug 16;7(Suppl 2):S4. doi: 10.1186/1471-2148-7-S2-S4 (PMC1963479; doi:10.1186/1471-2148-7-S2-S4)
Supplement: Additional file 5 — HIS4 multialignments with concatenated prokaryotic sequences. A multialignment of fungal HIS4 sequences and the corresponding proteins from Plants and from a selected number of Prokaryotes. [file 1471-2148-7-S2-S4-S5.pdf]

```
                                                      10        20        30        40        50        60        70
                                              ....|....|....|....|....|....|....|....|....|....|....|....|....|....|
10177677_A.thaliana                         1 ---------------------------------------------------------------------M 1
34904356_O.sativa (japonica cu              1 -------------------------------------------------------------MAAPPLPRA 9
10383761_S.cerevisiae                       1 ----MVLPILPLIDDLA------SWNSKKEYVSLVGQVLLDGSSLSNEEILQFSKEEEVPLVALSLPSGK 60
38567265_N.crassa                           1 METTLPLPFLVGVSVPPGLNDIKEGLSREEVSCLGCVFFEVKPQTLEKILRFLKRHNVEFEPYFDVTALE 70
50285163_C.glabrata                         1 ----MVFPILPVFGKDL------N-LASRPYIKLVNQAVLD-YTVSKEDIIKFVKDSQLLFVGTLLSSGK 58
42547615_G.zeae PH1                         1 METTLPLPFLVSVASANDQPT-NDGLNRQEIALLGAPFYETSSKDWG-------RPSAGTNVHMDATGLS 62
50304609_K.lactis                           1 -----MLPVVPVFN-AV------NALKEKTYLYLSSQLVLDGKDMTKDDILEFVQNSHGQNISVLLKDAK 58
38109852_M.grisea 7015                      1 MESTLPLPFIVDASVN--LNG-EAGLSKEQLACLGTIFFEVTPQNLGDVRSFLQPGTSAFEPYLDVTQLE 67
40746471_A.nidulans FGSC A4                 1 ----MATPFLVSYDPA----SASGGLSLQQIAYFGRVLIKAT--DLAQAETFIRQNFRLLDIYVDATGIS 60
50258877_C.neoformans var. neo              1 ---MSTPPFLPLVTSQDTT-------LLPSLALITPVLIPSDHLEQIRQS--------LPANASYYVQAN 52
44985362_A.gossypii ATCC 10895              1 ----MSFPLLVLVADIA------T-VKEKPFVLLSGQLLLDGSQSVETVVGFVKAYTQLRAVSVAYKTDE 59
46099735_U.maydis 521                       1 -MQQLTTPLLPLVDAESIKGS----ALIDAVSRIAPVLVPQDLVSTLPA-----------NVAYLVLAEP 54
31095443_H.cylindrosporum                   1 ---MAT--FLPLLTEENLP-------LFNALNLIGPIVLDAQRSVSLPR---------TPQHSYLLVDNS 49
49645975_Y.lipolytica CLIB99                1 -----MFPLLPQVSSVT------VPAVAVAPGKVLVSILQSDVPEIAK---ELKKAFG-QAQFHIMADTD 55
3203_P.pastoris                             1 ----MTFPLLPAYASV--------AEFDNSLSLVGKAVFPYAADQLHN---LIKFTQSTELQVNVQVESS 55
3757752_C.albicans                          1 ----MIFPILPVISSPE------DKQSIDEFSLVGQVLFPIESVSPKK---HFIHQFPHDLDIFVNAIDN 57
49656425_D.hansenii CBS767                  1 ----MTFPILPIISSS-------NKEAIAEFSVVGQILLPLESVSISK---HFLHQFPNYIDVNVDTTSS 56
7630171_S.pombe                             1 ---MALLPFFDLTNFES-----DASEELGWLKYVGRVQTRVFPQHFKDNLEKVRKISETIDVIVDTTAEL 62
N.mengitidis_MC58                           1 ---------------------------------------------------------------------- 1
15606967_A.aeolicus VF5                     1 ---------------------------------------------------------------------- 1
16080539_B.subtilis                         1 ---------------------------------------------------------------------- 1
15805759_D.radiodurans R1                   1 ---------------------------------------------------------------------- 1
16129967_E.coli K12                         1 ---------------------------------------------------------------------- 1
39996631_G.sulfurreducens PCA               1 ---------------------------------------------------------------------- 1
32265948_H.hepaticus ATCC 5144              1 ---------------------------------------------------------------------- 1
15673197_L.lactis subsp. lacti              1 ---------------------------------------------------------------------- 1
15841059_M.tuberculosis CDC155              1 ---------------------------------------------------------------------- 1
17230755_N.sp. PCC 7120                     1 ---------------------------------------------------------------------- 1
15925662_S.aureus subsp. aureu              1 ---------------------------------------------------------------------- 1
16332335_S.sp. PCC 6803                     1 ---------------------------------------------------------------------- 1
15643793_T.maritima MSB8                    1 ---------------------------------------------------------------------- 1
46199382_T.thermophilus HB27                1 ---------------------------------------------------------------------- 1
34556543_W.succinogenes DSM 17              1 ---------------------------------------------------------------------- 1
15669621_M.jannaschii DSM 2661              1 ---------------------------------------------------------------------- 1
15678273_M.thermautotrophicus               1 ---------------------------------------------------------------------- 1
20094973_M.kandleri AV19                    1 ---------------------------------------------------------------------- 1
20089786_M.acetivorans str. C2              1 ---------------------------------------------------------------------- 1
18312308_P.aerophilum str. IM2              1 ---------------------------------------------------------------------- 1
15897517_S.solfataricus P2                  1 ---------------------------------------------------------------------- 1
```

```
                                               80        90       100       110       120       130       140
                                      ....|....|....|....|....|....|....|....|....|....|....|....|....|....|
10177677_A.thaliana            2      AVSYNALAQSLA---------RSSCFIPKP---------------------------------------- 22
34904356_O.sativa (japonica cu 10     PVSSSAAAASPG---------GRALLLLRVGGGGSGRCAGVAAA-------------------------- 44
10383761_S.cerevisiae          60     -FSDDEIIAFLN-------NGVSSLFIASQDAKTAEHLVEQLNVPKERVVVE------------------ 104
38567265_N.crassa              70     --SIDDIITLLD-------AGARKVFVKTEQ---LADLSAYGSRVAPIVTGSSAALLSSATESGLLLSGF 128
50285163_C.glabrata            58     -FKDDEVIEILN-------NGVAVLFIEDHS--YAGYLVTEVGIPQERISVL------------------ 100
42547615_G.zeae PH1            62     --DPDDVVALLD-------SGVRTVFVISES---YSEYEQYGARVIPAVSS---LILSSATEHGLLVKDF 117
50304609_K.lactis              58     -FEDDDLIVLLN-------NGVVTLFIDSDD--YAAHLVEIG-VPSIRLTLL------------------ 99
38109852_M.grisea 7015         67     --SANDILSLLD-------SGARKVFVKPEQ---LKDYEEHGSRVGQAVDG--TSLQVSAAENGLLVSGI 123
40746471_A.nidulans FGSC A4    60     --ATGDLVDILN-------AGAAKIFISLDQ---LNALSEEQSVPSSRLVVYTSSNDQVEAFQKWVVKHI 118
50258877_C.neoformans var. neo 52     --DNDDLIALLD-------GGAEKLVVTPQQLGAGGAGIPKERLILQVSEEELST-SKRFAQQTGGILII 112
44985362_A.gossypii ATCC 10895 60     SYTVDDAFTMLN-------NGVSTLFVDERT----ADMLVDAGIPHTRLTVS------------------ 100
46099735_U.maydis 521          55     SLSYDAAADLLD-------AGAEAIVTADTQLIQQFDATIASARFIYLCASGAAPVAEVLKSVAGAVLTL 117
31095443_H.cylindrosporum      50     PVDLQDIASWLD-------EGVEKVIVPLASVKELIGVIPPDRLILLLDVANVSAFSEKVRNGVSGVLLK 112
49645975_Y.lipolytica CLIB99   55     -LSTDEYIHLLD-------AGASSVFVSGES---AVDLAASG-VPAGRLTVIGSKPDNLDVGGVFLKDTP 113
3203_P.pastoris                55     -VTEDQFEELIDNLLKLYNNGINEVILD-LD---LAERVVQRMIPGARVIYRTLVDK---VASLPANASI 117
3757752_C.albicans             57     -ATTDQIVELLN-------VGIKQVFVNEKQ---YHDAIEAG-SPSSRFVVAVDVP-----STELLTSEA 110
49656425_D.hansenii CBS767     57     KVDIDQIVELLN-------SGIKQVFIKESQ---IEEFILSGGLPSSRFAIKFDSSK---VSDTALETDS 113
7630171_S.pombe                62     --GPEACVNLLN-------AGALAILVNEEMLNELADISPNRLVLKTDTTDIGKIEKLSQVAGSIQWIGS 123
N.mengitidis_MC58              1      ---------------------------------------------------------------------- 1
15606967_A.aeolicus VF5        1      ---------------------------------------------------------------------- 1
16080539_B.subtilis            1      ---------------------------------------------------------------------- 1
15805759_D.radiodurans R1      1      ---------------------------------------------------------------------- 1
16129967_E.coli K12            1      ---------------------------------------------------------------------- 1
39996631_G.sulfurreducens PCA  1      ---------------------------------------------------------------------- 1
32265948_H.hepaticus ATCC 5144 1      ---------------------------------------------------------------------- 1
15673197_L.lactis subsp. lacti 1      ---------------------------------------------------------------------- 1
15841059_M.tuberculosis CDC155 1      ---------------------------------------------------------------------- 1
17230755_N.sp. PCC 7120        1      ---------------------------------------------------------------------- 1
15925662_S.aureus subsp. aureu 1      ---------------------------------------------------------------------- 1
16332335_S.sp. PCC 6803        1      ---------------------------------------------------------------------- 1
15643793_T.maritima MSB8       1      ---------------------------------------------------------------------- 1
46199382_T.thermophilus HB27   1      ---------------------------------------------------------------------- 1
34556543_W.succinogenes DSM 17 1      ---------------------------------------------------------------------- 1
15669621_M.jannaschii DSM 2661 1      ---------------------------------------------------------------------- 1
15678273_M.thermautotrophicus  1      ---------------------------------------------------------------------- 1
20094973_M.kandleri AV19       1      ---------------------------------------------------------------------- 1
20089786_M.acetivorans str. C2 1      ---------------------------------------------------------------------- 1
18312308_P.aerophilum str. IM2 1      ---------------------------------------------------------------------- 1
15897517_S.solfataricus P2     1      ---------------------------------------------------------------------- 1
```

```
                                            150       160       170       180       190       200       210
                                   ....|....|....|....|....|....|....|....|....|....|....|....|....|....|
10177677_A.thaliana          22   -------------------------------------YSFRDTKLRSRSNVVFACNDNKNIALQ------ 49
34904356_O.sativa (japonica cu 44   -----------------------------------AAPGWRRPFPAASVAARSAGTTPGEVAVD------ 73
10383761_S.cerevisiae        104  ---------------------------------ENGVFSNQFMVKQKF-S-QDKIVSIKKLSKD------ 133
38567265_N.crassa            129  DQTASEAAQFLEEARDKKITP-------FFIKPVPGADLEQFIQVAAKAN-AIPILPSTGLT----TKKD 186
50285163_C.glabrata          100  ---------------------------------NENVYETHFVIVNKFNA-KTQLVPVEEITSS------ 130
42547615_G.zeae PH1          118  DVSSSDVDKFIEVAQSKQIKS-------LYVKPTPETDIEKFIEVAKKAN-AIPIIPSTRLT----TDKN 175
50304609_K.lactis            99   ---------------------------------KDGAYQFSFG-QIQEIQ-QSQLVKASEISLE------ 128
38109852_M.grisea 7015       124  DASG-DVSTLVQQFNSKKGSP-------LFIRPADGADLELCAALARQVH-ATVILPSSRLTAC--TKDA 182
40746471_A.nidulans FGSC A4  119  EREEAGLCTDSAVVHSISVKLGLNPEAQLLYRTYSGDVTEDAVKDTMKQG-GVSIVPAAALT-I--SREE 184
50258877_C.neoformans var. neo 113  S----------CVPHNAKSLALPG---VDVFLQLPEVQPLRILNLIRSSRPSSYVIPSSYLSIESSTTAE 169
44985362_A.gossypii ATCC 10895 100  ---------------------------------DGSVYRQQYP-AVREFS-ERQIHSLSKVTGE------ 129
46099735_U.maydis 521        118  PAASAIASSSQLIKTTADVLQTKTSGKSLFVLATSGVPTVQDVQAVAQLT-ASLIAPSSVLGVAADEAHK 186
31095443_H.cylindrosporum    113  TPSIDFD---FISSISKFFAGST---IHVLSTSAILPTPANIRELRSIG-ATLIIPESQLTLAP-TSAT 173
49645975_Y.lipolytica CLIB99 114  ALEDVATLASTFAKQLLPSGG-------APVVYVN-VEDPKAAADLAATG-ATVVVSSAKLTTEH-QADK 173
3203_P.pastoris              118  AVPFSSPLG--DLKSFTNGGS-------RTVYAFSETAKLVDVTSTVASG-IIPIIDARQLTTEY-ELSE 176
3757752_C.albicans           111  SFVTSKPFSESDLKKYNANEN-------RVIYIES-NFTQDGAIELAKN--YVPVIPSTKLTVK----RE 166
49656425_D.hansenii CBS767   114  AFVFNCELAKDDAKKYSQSGN-------RGLYYENPSITQELANKLATED-IYPIIASESLTTA----SS 171
7630171_S.pombe              124  AENYPPDFFERASKIIHKAVMP-EGGGRTLYLEFPEQPSMEVLKSFSVHS-VVPVLSSSFLTVK---PAE 188
N.mengitidis_MC58            1    ---------------------------------------------------------------------- 1
15606967_A.aeolicus VF5      1    ---------------------------------------------------------------------- 1
16080539_B.subtilis          1    ---------------------------------------------------------------------- 1
15805759_D.radiodurans R1    1    ---------------------------------------------------------------------- 1
16129967_E.coli K12          1    ---------------------------------------------------------------------- 1
39996631_G.sulfurreducens PCA 1    ---------------------------------------------------------------------- 1
32265948_H.hepaticus ATCC 5144 1    ---------------------------------------------------------------------- 1
15673197_L.lactis subsp. lacti 1    ---------------------------------------------------------------------- 1
15841059_M.tuberculosis CDC155 1    ---------------------------------------------------------------------- 1
17230755_N.sp. PCC 7120      1    --------------------------------------------------------------------MS 2
15925662_S.aureus subsp. aureu 1    ---------------------------------------------------------------------- 1
16332335_S.sp. PCC 6803      1    -----------------------------------------------------MSSRSAGRNSPILPLMS 17
15643793_T.maritima MSB8     1    ---------------------------------------------------------------------- 1
46199382_T.thermophilus HB27 1    ---------------------------------------------------------------------- 1
34556543_W.succinogenes DSM 17 1    ---------------------------------------------------------------------- 1
15669621_M.jannaschii DSM 2661 1    ---------------------------------------------------------------------- 1
15678273_M.thermautotrophicus 1    ---------------------------------------------------------------------- 1
20094973_M.kandleri AV19     1    -----------------------------------------------------------------MSHVP 5
20089786_M.acetivorans str. C2 1    ---------------------------------------------------------------------- 1
18312308_P.aerophilum str. IM2 1    ----------------------------------------------------------------MEARPL 6
15897517_S.solfataricus P2   1    ---------------------------------------------------------------------- 1
```

```
                                               220       230       240       250       260       270       280
                                      ....|....|....|....|....|....|....|....|....|....|....|....|....|....|
10177677_A.thaliana            49     -----AKVDNLLDRIKWD-DKGLAVAIAQN-VDTGAVLMQGFVNREALSTTISSRKATFFS-RSRSTLWT 111
34904356_O.sativa (japonica cu 73     -----PKVEAILDSVKWD-SKGLAVAIAQN-VDTGAILMQGFANKEALATTISTRKATFYS-RSRSSLWT 135
10383761_S.cerevisiae          133    -----MLTKEVLGEVRTDRPDGLYTTLVVD--QYERCLGLVYSSKKSIAKAIDLGRGVYYS-RSRNEIWI 195
38567265_N.crassa              187    EAGKLAISTILSSVWKSDRPDGLLPTVVVD--EHDTALGLVYSSAESVNEALRTQTGVYQS--RKRGLWY 252
50285163_C.glabrata            130    -----TLSQTLIDSLKTDRADGLYTTLVVD--SQERCLGLVYSSKESIQMAINEESGIYYS-RSRDEIWV 192
42547615_G.zeae PH1            176    DSSRLLLSKLIASYWKSDRTDGLIPTVVTD--DAGIALGLAYTSEESILEALRTQTGVYQS--RKRGLWV 241
50304609_K.lactis              128    -----TFTNSVLSGMKTDRPDGLYTTLVVD--ENERSLGLVYSNKESVSLAIETQTGIYFS-RSRNEIWR 190
38109852_M.grisea 7015         183    TGGKVSISKLLASNWTSDRGDKLLPTVVTD--DNGIALGLVYSSEESIGEALRTCTGVYQS--RKRGLWY 248
40746471_A.nidulans FGSC A4    185    SSGKIQAGSLIAARGVKDQGNGLYATTVTD--ERGTCLGFVWSSDESIAEALRTGTGVYQS--RKRGLWY 250
50258877_C.neoformans var. neo 170    K---ISIPEAFLAPIISDRPDGLFPTIVSSYSHSTTPLGLVYSSIESVKESILTQKGVYQS--RKHGLWR 234
44985362_A.gossypii ATCC 10895 129    -----DFANTIISALKTDRADGLFSTLVVD--VYERCLGLVYSSEESIRLAIEKKAGVYYS-RSRNEVWE 191
46099735_U.maydis 521          187    QQGKLDIVDAFVAPLTSDRTDGLFATTVVS-SACTASLGLVYSSPLSIRKSIVTGSAHYQS--RNRGLWH 253
31095443_H.cylindrosporum      174    Q---LNIGDAFLAPVVTDRADGLFPTMVT--SETGHSLGLVYSSRESCKGKHNHRERYIPS--RKHGLWR 236
49645975_Y.lipolytica CLIB99   174    LDDKFPIADLLLASLSTDRQDGLYTTLVTD--VLNQSLGLVYSSAESIKEAIRTGTGVYQS--RRHGLWY 239
3203_P.pastoris                177    DVKKFPVSEILLASLTTDRPDGLFTTLVAD--SSNYSLGLVYSSKKSIPEAIRTQTGVYQS--RRHGLWY 242
3757752_C.albicans             167    EENKISISAVFVSTLTTDRPDGLYTTLITTPSPSYTALGIVYSSKDSIIAAIEEKVGVYQSRKRRDELWY 236
49656425_D.hansenii CBS767     172    ESGKISISSLFTAGLTTDRPDGLFTTLITTPAPSYTALGIVYSSVESIHASIAEKQGVYQSRKRRDELWY 241
7630171_S.pombe                189    EPKKLSLADLILISANTDREDGLFSTLVVN--ELGIALGLVYSSKESVAESLKTGTGVYQS--RKRGLWY 254
N.mengitidis_MC58              1      -------MDKNLLEAVKFDEKGLVCAIAQD-AETKRILMVAWMNAEALQKTVETGFAHYYS-RSRQKQWM 61
15606967_A.aeolicus VF5        1      ------------MLKLKFNEEGLIPVIAQD-YRTGEVRMLAYANEEAIKKTLETGYAHYYS-RSRKKIWK 56
16080539_B.subtilis            1      ---------MKQADELRFNEDGLIPAIVQD-AASKEVLTLAYMNKESYEKTLETKETWFYS-RSRQALWH 59
15805759_D.radiodurans R1      1      ----MTDLS-----ELNFDPSGLIPVVTQD-ARSGAVLMQAYADRAAVERTLDTREATYYS-RSRGEQWV 59
16129967_E.coli K12            1      -----MLTEQQRRELDWEKTDGLMPVIVQH-AVSGEVLMLGYMNPEALDKTLESGKVTFFS-RTKQRLWT 63
39996631_G.sulfurreducens PCA  1      ------------MITIDFQKMGGLIPAIIQDHATNEVLMVAFMDEKTLNLTLESGKTWFFS-RSRNKYWM 57
32265948_H.hepaticus ATCC 5144 1      --------MQDVFRQIDWERYELIPTIVQE-KQSQQILMLAYSSKQSLELSLQTHLAHYFS-RSKQRIWQ 60
15673197_L.lactis subsp. lacti 1      ---------MRPD----FHKQELIPVIVQD-YQTNQVLMLAYTNEVAFEKMLETGETWFWS-RSRQKLWH 55
15841059_M.tuberculosis CDC155 1      -----MTLDPKIAARLKRNADGLVTAVVQE-RGSGDVLMVAWMNDEALARTLQTREATYYS-RSRAEQWV 63
17230755_N.sp. PCC 7120        3      FIDSLSPQNVIPVEEIRYDERGLVPAIVQD-YLDGTVLMMAWMNRESLQKTLDTGETWFWS-RSRQEFWH 70
15925662_S.aureus subsp. aureu 1      ----------MTKYKIDFS-KGLVPAILQD-NQTKQVLMLGYMNQEAFDKTIEDGVVCFYS-RSKQRLWT 57
16332335_S.sp. PCC 6803       18     HSD-LPLANAVPLDKIRYNDQGLVPAIAQD-YLDGTVLMLAWMNEAALAKTLATGQVWYWS-RSRQELWH 84
15643793_T.maritima MSB8       1      -------------------MMTLYPVVVQE-RTTGEVLMLAYANEEALELTKKTGYAHFFS-RERQKIWK 49
46199382_T.thermophilus HB27   1      -----MDLS-----AVRFDEKGLVPVVVQD-ARTGEVLTLAYANREALEETLRTRRSTFFS-RSRQALWR 58
34556543_W.succinogenes DSM 17 1      --------MNTLLDGIDWEKHPLLPAIVQE-RGSGEVLMLAYMNQEALNLTLSTQVAHYFS-RSKGRIWK 60
15669621_M.jannaschii DSM 2661 1      MDVEDT-VKKLNLKFR-NIEGERLILAITCD-ENKNVLMVAFMNEEALKKTLETGYMHYYS-TSRKKLWR 66
15678273_M.thermautotrophicus  1      MIKSKG-DVNILLNFRHNINGEDLIIAVAQDHETGEVLMVAYMNREALRRTLETGTAHYWS-TSRGKLWL 68
20094973_M.kandleri AV19       6      LDPETARKVTSSLNYRFEIGGEPLVIAIAQDAENGDVLMTAFANEEAVYRTLTTGYAHYWS-TSRREVWK 74
20089786_M.acetivorans str. C2 1      ---------MIDLDTLKYENG--LILAVVQDQKSREVLMCAYMNREALEKTVKTGIAHFWS-RSRKQLWK 58
18312308_P.aerophilum str. IM2 7      ATPEEAWRIASSLRYRHIEGT---VVAVVQDVETKEVLMVGHMDPIAVVLTLTTGLAHYYS-TTRKRIWL 72
15897517_S.solfataricus P2     1      --------------------------------------MVGNMNREALFKTLTTGYLHFWS-LSRKKLWL 31
```

```
                                                      290       300       310       320       330       340       350
                                                 ....|....|....|....|....|....|....|....|....|....|....|....|....|....|
10177677_A.thaliana                          112 KGETSNNFINILDVYVDCDRDSIIYLGTPDGPTCHTGEETCYYTSVFDQLNNDEA--------------- 166
34904356_O.sativa (japonica cu               136 KGETSMNFINVHDIFLDCDRDSIIYLGKPDGPTCHTGAETCYYTSVYDALQGSKP--------------- 190
10383761_S.cerevisiae                        196 KGETSGNGQKLLQISTDCDSDALKFIVEQE------NVGFCHLET-MSCFGEF----------------- 241
38567265_N.crassa                            253 KGATSGDTQELVRISLDCDNDALKFVVKQ------KGR-FCHLDQ-SGCFGQL----------------- 297
50285163_C.glabrata                          193 KGATSGNTQKLLSISVDCDGDALKFVVDQQG-K---DRSFCHLNT-ESCFGGF----------------- 240
42547615_G.zeae PH1                          242 KGLTSGDTQELLRIGLDCDNDTIKFVVNQ------KGR-FCHLQQ-FGCFGDL----------------- 286
50304609_K.lactis                            191 KGATSGNVQKLLSIELDCDGDALKFVVRQGG-----SGSFCHLET-ESCFGNF----------------- 237
38109852_M.grisea 7015                       249 KGATSGDTQELVRISLDCDNDALKFVVRQ------KGR-FCHLDQ-FSCFGNL----------------- 293
40746471_A.nidulans FGSC A4                  251 KGQSSGDVQELIRIGFDCDSDCLVFIVKQ------IGRGFCHLGT-ASCFGPY----------------- 296
50258877_C.neoformans var. neo              235 KGETSGAVQQVTGIKLDCDNDALIFEVVQHG------SGFCHLP-QSTCFGNL----------------- 280
44985362_A.gossypii ATCC 10895               192 KGRTSGNTQELLQVAIDCDGDALRYVVDQRS-EQGGMGNFCHLNT-PSCFGSL----------------- 242
46099735_U.maydis 521                        254 KGESSGATQQVVSIRQDCDSDAIQFAVRQSR-GT-LASGFCHLENREGCFSSA----------------- 304
31095443_H.cylindrosporum                    237 KGETSGATQDVVRIRLDCDSDSLEFRVVQHG------AGFCHLN-RRSCFSEA----------------- 282
49645975_Y.lipolytica CLIB99                 240 KGKESGNTQKLIRIETDCDGDCLKFIVEQ------TGAGFCHFNT-ESCFSA------------------ 284
3203_P.pastoris                              243 KGATSGATQKLLGIELDCDGDCLKFVVEQ------TGVGFCHLER-TSCFGQ------------------ 287
3757752_C.albicans                           237 KGKTSGATQKLVKLSKDCDSDVIQFMVEPR-----TGYGFCHRETKFTCFGD------------------ 283
49656425_D.hansenii CBS767                   242 KGKTSGATQKLVKLEKDCDSDVVKFIVEPR-----EGYGFCHLDKNFTCFHDGELK-------------- 292
7630171_S.pombe                              255 KGASSGAVQHLIHIDVDCDEDCLRFVVYQT------GKGFCHLDT-LHCFGQA----------------- 300
N.mengitidis_MC58                             62 KGEESGHTQKVRALRLDCDGDAIVMLIA------QNGGIACHTGR-ESCFYKVWRGSAWETADAVL---K 121
15606967_A.aeolicus VF5                       57 KGETSGELQKVIEVRVDCDEDALIYVIE------QEKDRACHTGE-RNCFFRDIEGNK--VEKPLP---F 114
16080539_B.subtilis                           60 KGETSGNTQAVKGIRYDCDQDALLVLVE-------PSGPACHTGS-YSCFTKEQTEEQAADRF------- 114
15805759_D.radiodurans R1                     60 KGQTSGHTQRVVSVHVDCDGDSLLYRVE-------QTGPACHTGE-YSCFYRPLLE--DDAPDT------ 113
16129967_E.coli K12                           64 KGETSGNFLNVVSIAPDCDNDTLLVLANPIG-------PTCHKGT-SSCFGDT----------------- 108
39996631_G.sulfurreducens PCA                 58 KGEESGNTQEVVEVLTDCDADAVVIKVK------QNGPAACHTGN-RSCFYVRWEDGQWVEHSEPL---F 117
32265948_H.hepaticus ATCC 5144                61 KGEQSGHIQHIKEVKLDCDNDSLIFIVE------QVG-VACHTGE-KSCFFRIFSLDKNCQNPPVS---M 119
15673197_L.lactis subsp. lacti                56 KGEESGHFQKIKGMRLDCDQDTLLVFVE-------QIGNACHTGT-YSCFYDELIPFDDSD--------- 108
15841059_M.tuberculosis CDC155                64 KGATSGHTQHVHSVRLDCDGDAVLLTVD-------QVGGACHTGD-HSCFDAAVLLEPDDMQQS------ 119
17230755_N.sp. PCC 7120                       71 KGGTSGHTQKVQSIRYDCDSDALLVGVE------QIGDIACHTGE-RSCFHQVEGKIVAPPGD------- 126
15925662_S.aureus subsp. aureu                58 KGETSGHTQLVKDIHVDCDNDTILIDVI-------PNGPTCHTGS-QSCFNTEVPFS------------- 106
16332335_S.sp. PCC 6803                       85 KGATSGHFQKLLGIRYDCDSDALLLTIE------QKGDIACHTGE-RSCFHQLDGHKSPPPAD------- 140
15643793_T.maritima MSB8                      50 KGETSGNTMRVVEIRRDCDDDAYLYIVDF-----PEDKVACHTGN-RSCFFKVEHRFEETGSPTFW---L 110
46199382_T.thermophilus HB27                  59 KGETSGHTQEVVEVLLDCDGDAVVYRVL-------PQGPACHTGE-RTCFHRALLE--GEK--------- 109
34556543_W.succinogenes DSM 17                61 KGESSGHIQKIHEIFLDCDSDTILLQVE------QVG-VACHTGR-KSCFFQKVELDS---SLSLT---S 116
15669621_M.jannaschii DSM 2661                67 KGEESGNVQKLIKFYRDCDGDALLFIVE-------QKGVACHEGY-YSCFHYKIE-DGELKITG------ 121
15678273_M.thermautotrophicus                 69 KGESSGHVQRVKDVLVDCDGDAVVLKVE-------QEGGACHTGY-RSCFYRSID-GDELKVREDAVKVF 129
20094973_M.kandleri AV19                      75 KGEESGHVQRVVEVRVDCDKDAVLYVVE-------QEGGACHTGY-RSCFYRRVTRDGSFEVVMDR--VF 134
20089786_M.acetivorans str. C2                59 KGETSGHLQKVKEIRIDCDMDSVLLLVE-------QVGGACHMGY-RSCFYRNLK----GEVVGEK--VF 114
18312308_P.aerophilum str. IM2                73 KGETSGHYQIVKEFRSDCDGDAVVIKVV-------QIGAACHTGS-RSCFESKYS-----LIKA------ 123
15897517_S.solfataricus P2                    32 KGETSGNFQIIEEFKVDCDADAVLFKVT-------SLGPICHTGN-YTCFYRSYD-----ELVN------ 82
```

```
                                              360       370       380       390       400       410       420
                                          ....|....|....|....|....|....|....|....|....|....|....|....|....|
10177677_A.thaliana                 166   ------SGNKLALTTLYSLESIISKRKEESTVPQEGKPSWTRRLLTD-DALLCSKIREEADELCRTLEDN 229
34904356_O.sativa (japonica cu      190   ------NQDRQVVSTLYSLEDTISRRKEEIVTEGSGKPSWTKKLILD-NRLLCSKISEEAGELNQTLLEN 253
10383761_S.cerevisiae               241   ------------KHGLVGLESLLKQRLQD-----APEESYTRRLFND-SALLDAKIKEEAEELTEAKG-- 291
38567265_N.crassa                   297   -------------KGLPKLEQTLISRKQS-----APEGSYTARLFSD-EKLVRAKIMEEAEELCTAQT-- 346
50285163_C.glabrata                 240   ------------THGLASLEQLLRDRFES-----APEGSYTKRLFND-PELLNAKIKEEAEELTEAED-- 290
42547615_G.zeae PH1                 286   -------------NGISALEQTLKSRKES-----APEGSYTARLFSD-EKLLRAKIMEEAEELCDGKT-- 335
50304609_K.lactis                   237   ------------RHGLYGLQKLLQERLLN-----APEGSYTKRLFND-SDLLTAKIKEEAEELTEAVD-- 287
38109852_M.grisea 7015              293   -------------GGIAKLEQTLTQRRES-----APAGSYTARLFSD-EKLLRAKIMEEAEELCDAKT-- 342
40746471_A.nidulans FGSC A4         296   -------------TGLSRLQKTLQARKAD-----APAGSYTARLFNE-PKLTQAKIMEEADELCRAET-- 345
50258877_C.neoformans var. neo      280   -------------SGIAKLSDTLTSRLAS-----APEGSYTKRLFTD-EKLLRSKIMEEAEELCDAQT-- 329
44985362_A.gossypii ATCC 10895      242   ------------RRGLESLEQIVDQRIAD-----ARPESYTLRLVGD-TELLNSKIKEEADEVIEAIT-- 292
46099735_U.maydis 521               304   -------------TGLAKLEATLKHRLQT-----APAGSYTARLFND-ASLLGAKLREEAAELADANNN- 354
31095443_H.cylindrosporum           282   -------------SGLSLLEATLISRLES-----APEGSYTKRLFND-ANLLQSKIMEEADELCRATT-- 331
49645975_Y.lipolytica CLIB99        284   ------------AQGISCLEKTLQQRLQN-----APAGSYTKRLFDD-SDLLRAKIMEEAEELVEA---- 332
3203_P.pastoris                     287   ------------SKGLRAMEATLWDRKSN-----APEGSYTKRLFDD-EVLLNAKIREEADELAEA---- 335
3757752_C.albicans                  283   ------DIADSPARGLPKLDSTLQDRLEN-----APEGSYTKRLFDD-EKLLIAKLKEELDELIEA---- 337
49656425_D.hansenii CBS767          292   ------QKGESFGKGLARLDNTLQDRFQS-----APEGSYTKRLFND-EPLLIAKLKEELDELIEAGQSS 350
7630171_S.pombe                     300   -------------SGLCQLEKTLIDRKNN-----APEGSYTARLFSD-PKLLRAKIMEEAEELCDATT-- 349
N.mengitidis_MC58                   122   DEKEIYGSTHMGDSVLSAIQQTITQRKSANPSESYVAQLLHKGE-----DKILKKVIEEAGEVLMASKDK 186
15606967_A.aeolicus VF5             115   E-------------VLPRLQDVIREKIERKEENSYTAKLVSQGK-----ERVFQKFGEEAVETLIALMKG 166
16080539_B.subtilis                 114   -------------GIMNELERVIAERQAEMPEGAYTTYLFREGV-----DKILKKVGEEASEVIIAAKNR 166
15805759_D.radiodurans R1           113   ----------GLDGTLERVYATITERLATLPEGSYVARLHAGGL-----DRVLKKISEEAGEVLLAAKNA 168
16129967_E.coli K12                 108   ---------AHQWLFLYQLEQLLAERKSA-----DPETSYTAKLYASGTKRIAQKVGEEGVETALAATVH 164
39996631_G.sulfurreducens PCA       118   DPAEVYKKMNERDDILQAVYEVILDRKGALPESSYTASLFHKGI-----DKILKKVGEEATEVIIAGKGG 182
32265948_H.hepaticus ATCC 5144      120   PQKYPIGVYH----ILDDLYHIIEQRRCENIEHSYTASLLAKGV-----NGIGKKIIEEAGELCFALKDK 180
15673197_L.lactis subsp. lacti      108   --------------IFSELEKQIIDRKLHPVEKSYTNYLLGEGI-----DKVLKKVGEEASEVIIASKNS 159
15841059_M.tuberculosis CDC155      119   ----------LAVKTFEDLFAELGDRARTRPADSTTVAALDGGV-----HALGKKLLEEAGEVWLAAEHE 174
17230755_N.sp. PCC 7120             126   --------------TLSQVFQVICDRRNHPTESSYTSKLFAGGD-----NKILKKIGEESAEVVMACKDD 177
15925662_S.aureus subsp. aureu      106   ---------------VQTLAQTVQDSAQSNNEKSYTKYLLTEGI-----EKITKKYGEEAFEVVIEAIKG 156
16332335_S.sp. PCC 6803             140   --------------MLTELARVIGDRRDHPTPESYTCKLLAGGD-----NKILKKIGEESAEVVMACKDD 191
15643793_T.maritima MSB8            111   E----------------LYRLVRKRKEEMPEGSYTVKLFKEGK-----GKIAKKFGEEAVEVITGYLQN 158
46199382_T.thermophilus HB27        109   ----------DLGFVVGQVYATIKERLRTLPEGSYVARMHHAGL-----DRILKKIGEEAGEVILAAKNQ 164
34556543_W.succinogenes DSM 17      117   EAPDTSALYG----VVDRLYHELLARQGADPQSSYTAKLFSKGE-----NTIGKKIVEEAAELSFAIKDS 177
15669621_M.jannaschii DSM 2661      121   ---EYYSKRMI----LEEVYEIIKQRIKEKPEGSYVAKLTTDDK-KTAINKICEKIGEESTELILAAKDD 183
15678273_M.thermautotrophicus       130   DPEEIYGDGMVDEGILREVYRVLEDRR-DRPIDSYTSRLMRDDD-KMAEDKILEKIGEEAAEVIIASKND 197
20094973_M.kandleri AV19            135   NPDEVYRMGDPE--VLLEVYEVIRNRIEERPEGSYVAELTEDDDTKPAINKICEKIIEESGELILAAKDG 202
20089786_M.acetivorans str. C2      115   EPKDVYMPEADLS-ILNRVYDIILDRKENYDENSYVCKLLNHRK---GMNKILEKVGEESIETILAVRNE 180
18312308_P.aerophilum str. IM2      123   --LKLSLRHGMSCQIFEKLEAVIRQRIAEGNPQSYTYRLYSSGV-----STIARKVGEEAVEVAVAALAE 186
15897517_S.solfataricus P2          82    --SKS--MHDMSNEIVDELYKIILDRIEKRPTGSYTAEIVNKGK-----PYVARKVGEESVETIVASLAE 143
```

```
                                             430       440       450       460       470       480       490
                                    ....|....|....|....|....|....|....|....|....|....|....|....|....|....|
10177677_A.thaliana             229 -EEVSRTPSEMADVLYHAMVLLSKRGVKMEDVLEVLRKRFSQSGIEEKQNRTK---------------- 281
34904356_O.sativa (japonica cu  253 -EDESRTISEMGDLLYHAMVLLRVKGVRMEQVLEVLRKRFSQSGIEEKASRNKS--------------- 306
10383761_S.cerevisiae           291 ---KKELSWEAADLFYFALAKLVANDVSLKDVENNLNMKHLKVTRRKGDAKPKFVG------------- 344
38567265_N.crassa               346 ---PQEIAFEAADLFYFALTRAVAAGVTLADIERSLDAKSWKVKRRTGDAKGKWAEKEGIKP--AASALA 411
50285163_C.glabrata             290 ---KNEISWEAADLFYFALTKLVANNVSLKDVESNLNMKHLKITRRKGDAKDKFIKKQEP---------- 347
42547615_G.zeae PH1             335 ---KENIAFEAADLIYFALTKAVGAGVSLADIEANLDAKSLKVKRRTGNAKGKWAEKEGIKT---EETPA 399
50304609_K.lactis               287 ---KKDIAWECADLFYFAMARLVANGVSLEDVERNLNTKHLKITRRKGDAKPKFLK------------- 340
38109852_M.grisea 7015         342 ---KENVAFEAADLIYFALTKAVASGVSLSDIERNLDAKSWKVKRRTGDAKGKWAEKEGIKPS-APSALA 408
40746471_A.nidulans FGSC A4    345 ---KEDIAFEAADLLYFALTRCVAAGVSLEDVERNLDLKSLKVKRRKGDAKGPWAEKAGLAE--KPAEAK 410
50258877_C.neoformans var. neo 329 ---KEEVAFEAADLVYFALTRCVSKGVSWRDVEAALDKKALKVTRRKGDAKPKWEEKTREVVNENGEAKP 396
44985362_A.gossypii ATCC 10895 292 ---PAELQWEVADLLYFLMVKMRSNNVTLKEVEANLNMKHMKITRRPGNAKPKYLPAQEWQ--------- 350
46099735_U.maydis 521          354 --DKKHVAFEAADLIYFALTKCVSAGIGLEEIEASLDAKAKKVSRRKGDAKPAFAAQQK-----AAETQR 417
31095443_H.cylindrosporum      331 ---KEEIAFEAADLFYFALTKCVAAGVGVADIEHSLDRKAKRITRRTGNAKPQWTS---------GEST- 388
49645975_Y.lipolytica CLIB99   332 -KDAEEVSWEAADLIYFAMVKVVKEGVSWAQVMNNLDSKHMKISRRKGDAKQKWVDALGIKR--QKVDEK 399
3203_P.pastoris                335 -KSKEDIAWECADLFYFALVRCAKYGVTLDEVERNLDMKSLKVTRRKGDAKPGYTKEQPKE--------- 395
3757752_C.albicans             337 -KSKEEIAWECADLVYFAMVWCIKHGVRLADIEKNLDVKSLKVSRRKGDAKPQYQEAP----------- 394
49656425_D.hansenii CBS767     351 SKDTNEVAWECADLFYFAMVWCIKNGVRLADVERNLDIKSGKVTRRKGDAKPAYLKTDNE---------- 410
7630171_S.pombe                349 ---KENVIWEMADLMYFAITRCVGSGVSLNDISRHLDLKHRKVTRRKGDAKVAWQEKLKDKG-------- 408
N.mengitidis_MC58              187 N--PSHLVYEVADLWFHTMILLTHHDLKAEDVLDELARRQGLSGLVEKAARTES---MKKLN-------- 243
15606967_A.aeolicus VF5        167 E--KEEVIYESADMLYTFLVSLSVSGIDIKEVMEELIRRFKMR-------------IEDLR-------- 212
16080539_B.subtilis            167 D--HEELKWEAADLLYHLLVLLREQSLPLDDVLDVLKKRHS---------------EIEEMK-------- 211
15805759_D.radiodurans R1      169 D--RAELATETADLLFHTLFALAEVGVSPADVAAVLQGREG---------------KSGLKG------- 213
16129967_E.coli K12            164 --DRFELTNEASDLMYHLLVLLQDQGLDLTTVIENLRKRHQMSFN----------------------- 207
39996631_G.sulfurreducens PCA  183 K--REEIVYETADLLFHTLVLLGHYDIAPADVYNELRRRFGTSGHAEKASRNE----MRFLD-------- 238
32265948_H.hepaticus ATCC 5144 181 D--EKAIIYECADLFYHILVGLALEHITPERVLQELRRRMGQSGIEEKASRK----HMKYLD-------- 236
15673197_L.lactis subsp. lacti 160 D--KGELLGEIDDLLYHLFVLMNQQGISLEEVRQKAKERHQLEG------------NKKEFH-------- 207
15841059_M.tuberculosis CDC155 175 S--NDALAEEISQLLYWTQVLMISRGLSLDDVYRKL---MLT---------------RIDLRG------- 217
17230755_N.sp. PCC 7120        178 D--QEAIAGEVADLLYHTLVALAHHQVDIKAVYRKLQERRR---------------MLVLK-------- 221
15925662_S.aureus subsp. aureu 157 D--KKAFVSEVADELYHLFVLMHALGVDFSEIEAELARRHHKRNNFKGERQ-----NIEQWM-------- 211
16332335_S.sp. PCC 6803        192 D--PEAIAGEVADLFYHTLVALAHHNVDLRAVYRKLGDRRR---------------MTRIL-------- 235
15643793_T.maritima MSB8       159 D--RENLVWEIADMMYHLTVLMADAGVTVQDVMRELEKR-----------------RKMI-------- 199
46199382_T.thermophilus HB27   165 N--PEELRHEAADLLFHLLLTLAELGLTPEDLAKTLWERHRP---------------RSPYDG------- 210
34556543_W.succinogenes DSM 17 178 S--ESEIVYEAADLLYHALVGLAFRGIHPDKIKQELQRRQGVSGIAEKNSRKDSMRKIKRLN-------- 237
15669621_M.jannaschii DSM 2661 184 K--KDEIIYEAADLIFHTMVLLAYKNIEFEELLKEFERR-----------------KKMVTG------- 226
15678273_M.thermautotrophicus  197 ----ENLVEEAADLIFHTLLLLVYKGVPLDSLLEEFAAR-----------------RKMLM-------- 237
20094973_M.kandleri AV19       203 D--REGVVYESTDLIFHVLVLLAYLGIEIGEVFDEFERR-----------------RKMRL-------- 244
20089786_M.acetivorans str. C2 181 D--HKEIVSESSDLIFHLLVLLAANNVTLDEIAGELSARHEKM-------------KRDMVT------- 227
18312308_P.aerophilum str. IM2 187 G--RERVVEESADLLYHLLVLLNSLGLSLGDVCKELERR-----------------MKMRG-------- 228
15897517_S.solfataricus P2     144 N--KERFISEVADLIYHLLVLMALEGVTPEDIYRELERR-----------------RKMIS-------- 185
```

```
                                                 500       510       520       530       540       550       560
                                             ....|....|....|....|....|....|....|....|....|....|....|....|....|....|
10177677_A.thaliana                 281     --------------------MKSYRLSELSSSQVDSLKSRPRIDFS-----------------SIFATVN 314
34904356_O.sativa (japonica cu      306     --------------------MKSYRLSELSDAEVGGLKARPRIDFS-----------------SIFGTVN 339
10383761_S.cerevisiae               344     --------QPKAEEEKLTGPIHLDVVKASDKVGVQKALSRPIQKTS-----------------EIMHLVN 389
38567265_N.crassa                   412     ATSAPVTKEAAQETTPEKITMRRFDASKVSTEELDAALKRPAQKSSD----------------AIYKIIV 465
50285163_C.glabrata                 347     --------QAQNDEELSSKPIHLNVVKTSNKKEVEAALTRPIQKTS-----------------EIMHLVN 392
42547615_G.zeae PH1                 400     KAPQP----EAEKPADGRIAMERVDSTKISQADLVEKLKRPSQKSPD----------------AILKIIK 449
50304609_K.lactis                   340     --------KEPVAVHEEDGKIVLNVVSASDKAAVEKAVTRPIQKTA-----------------EIMNLVN 385
38109852_M.grisea 7015              409     PAPAP----AATEATSDRIAMKVLDVSQSSVADIQEALKRPSQKSSD----------------AIMKIIG 458
40746471_A.nidulans FGSC A4         411     PAPKP----EEPKEDTSRIEMTRVATASTPAEKVQEYLKRPSQKSND----------------AIVGLVK 460
50258877_C.neoformans var. neo      397     TVPEPTKLPE-TESEDVPIKMRAVTLSTLSVLEQKDLLLRPVLNSL-----------------AMIDKVK 448
44985362_A.gossypii ATCC 10895      350     -------KNKETPVDIAPSAIYLNVVSSDDEAALKTAITRPIQKTT-----------------DILGLVE 396
46099735_U.maydis 521               418     AAGTPAAAAETVKDASAPIKIQSYRYEDLSAAQRKELLKRPALRTE-----------------QVMDICR 470
31095443_H.cylindrosporum           389     AAPEKKETEK-GKVVDGPIRMRTSDLSTLSAQERAQLLRRPVLKSD-----------------EMIEKVK 440
49645975_Y.lipolytica CLIB99        400     PKEEKKEEKKEEIKEDGKIQLNHINADTASKAEIETVLARPAQKTS-----------------DIMKLVL 452
3203_P.pastoris                     395     ------ESKPKEVPSEGRIELCKIDVSKASSQEIEDALRRPIQKTE-----------------QIMELVK 442
3757752_C.albicans                  394     ------------VNSSYKLEIVSVDDAAA----VERAMTRPVQKTA-----------------DIMKLVL 431
49656425_D.hansenii CBS767          410     --------KSEQSNEEYKMETIYVNDKTTDPKDIAKALNRPVQKTA-----------------DIMKLVL 455
7630171_S.pombe                     408     ----GVANTSYTAMPEYEIQVPSYRAAALTAEERTRLLARPIQNTQ-----------------KIRTIVQ 457
N.mengitidis_MC58                   243     ------------------------TQSPDFQAGLKALLAFETAQNP-----------------ETERIVA 272
15606967_A.aeolicus VF5             212     ------------------------KEDWKLNERLEYLAKRGEILEE-----------------EYEKSVK 241
16080539_B.subtilis                 211     ------------------------IKTISGAERLSLKRSIDAGTE------------------EQRKTVR 239
15805759_D.radiodurans R1           213     --------------------PKEVGMQVLQGAEARAALTRTFSQIPVPDAVLSRIEQTFGERLTPEQVVE 263
16129967_E.coli K12                 207     ---------------------TIIDWNSCTAEQQRQLLMRPAISASE----------------SITRTVN 240
39996631_G.sulfurreducens PCA       238     ------------------------IRDTNFDAEFAAILARGEETGR-----------------EVEQVVL 267
32265948_H.hepaticus ATCC 5144      236     ------------------------VRLPEFANEFQQVLSRGKMDMK-----------------EVSSLVQ 265
15673197_L.lactis subsp. lacti      207     ------------------------TRTADMLKQIDYQGKLEEIAEKFQG-------RKTELSKEVNKTVQ 246
15841059_M.tuberculosis CDC155      217     --------------------------AELTAAELRAALPRGG-----------------ADVEAVLPTVR 244
17230755_N.sp. PCC 7120             221     ------------------------TTDKEFSPRFQSLVSDRREATV----------------DVS-GTVR 250
15925662_S.aureus subsp. aureu      211     ------------------------LNAQQFLNQFSLEAPLDESLY---------------------PIIR 236
16332335_S.sp. PCC 6803             235     ------------------------KLS-HLTPQQLNQLKRRSEQNI----------------DQALAIAK 264
15643793_T.maritima MSB8            199     ------------------------LMNNPGDKEVLRLLKQRMESVS-----------------QVEETVK 228
46199382_T.thermophilus HB27        210     ----------------------SHGNMIYAAEEVRARFARRG--------------LSFDP--TVEEIVR 242
34556543_W.succinogenes DSM 17      237     ------------------------SSLAEFRREFDELLLRGNVDMD-----------------TVIPVVS 266
15669621_M.jannaschii DSM 2661      226     --------------------MIIKKIKELTKEEEEKIINRNKANFE-----------------EILPTVM 259
15678273_M.thermautotrophicus       237     ------------------------KITEFDYRRISELVERARLDVD-----------------DVLGPVA 266
20094973_M.kandleri AV19            244     -------------------------LVRPEEEELERVLRRSEMDVT-----------------EVLPDVE 272
20089786_M.acetivorans str. C2      227     --------------------MLFKKLSDASEAEMKKLISRG-SGLA-----------------DVGETVS 259
18312308_P.aerophilum str. IM2      228     ----------------------------------FPR--------------------------EVLESVW 238
15897517_S.solfataricus P2          185     ----------------------------------YSLPNERPNDFS-----------------RVIPVVK 204
```

```
                                              570       580       590       600       610       620       630
                                      ....|....|....|....|....|....|....|....|....|....|....|....|....|....|
10177677_A.thaliana             315  PIIDAVRSNGDNAVKEYTERFDKVQL--NKVVEDMSELSVPELD---SNVKEAFDVAYDNIYAFHLAQKS 379
34904356_O.sativa (japonica cu  340  PIVEDVRMRGDAAVKDYTVKFDKVAL--DDVVVRVSDLPDVELD---PAVKEAFDVAYDNIYAFHVSQKL 404
10383761_S.cerevisiae           390  PIIENVRDKGNSALLEYTEKFDGVK---LSNPVLNAPFPEEYFEGLTEEMKEALDLSIENVRKFHAAQLP 456
38567265_N.crassa               466  PIIEDVRKNGDKAVLSYTHKFEKATS--LTSPVLKAPFPKELMQ-LPEETIAAIDVSFENIRKFHAAQK- 531
50285163_C.glabrata             393  PIIEGVRSKGDKALIEFTAKFDGVQ---LQSPVLEAPFPPEYFEGLTEEMKESLDLSIENVRKFHAAQLQ 459
42547615_G.zeae PH1             450  PIIEEVRTGGDKAVLSYTHKFEKATS--LTSPVLKAPFPKELMD-ISPETIEAIDISFENIKKFHSAQQ- 515
50304609_K.lactis               386  PIIENVIKNGDKALVELTAKFDGVQ---LETPVLEAPYPEEYLDGLTDELRDALDLSIENVKKFHAAQMQ 452
38109852_M.grisea 7015          459  PIVDDVHTNGDKAVLSYTHKFEKATS--LTSPVLKAPFPEEMMR-LSPETAKAIDISFENIRKFHAAQK- 524
40746471_A.nidulans FGSC A4     461  PIIQDVREQGDAGVLKYTHKFEKATS--LTSPVLKAPFPAELMK-LSPEVQEAIDVSISNIARFHSAQKG 527
50258877_C.neoformans var. neo  449  PIVERVRQEGDAGLKAMTKQFDRADL--SSNVLLPPFETPGEDVLPK-DVREAIDVAYNNVKEFHQAQN- 514
44985362_A.gossypii ATCC 10895  397  PIIKKVIEEGDNALTELTARFDGVK---IETPVLEAPFGDEYLKGLTEDVRTAIDISMENVRKFHAAQLR 463
46099735_U.maydis 521           471  PILRSVKERGDAALLELTEKFDKAKL--TNAVRLPPFVDDSVMAQIKPEVKVAIDIAYNNIYKFHKAQKT 538
31095443_H.cylindrosporum       441  PIVEAVRTRGDDALLEFTAKFDKAQL--TSTVLLPPFA-PESMVVEE-NVRKAIDVAYGNIRKFHEAQK- 505
49645975_Y.lipolytica CLIB99    453  PIVEGVKTGGDKKLLELTEKFDGVK---LESPILTAPFSEDLMK-ISDDVKASIDMSIANVKKFHAAQLD 518
3203_P.pastoris                 443  PIVDNVRQNGDKALLELTAKFDGVA---LKTPVLEAPFPEELMQ-LPDNVKRAIDLSIDNVRKFHEAQLT 508
3757752_C.albicans              432  PIIEKVKSDGDKALIELTSKFDGVK---LDAPVLQAPFPADLMD-ISEEMKAAIDLSMQNIEKFHAAQLP 497
49656425_D.hansenii CBS767      456  PIIDKVKKDGDKALLELTSKFDGVK---LKSPVLSAPFPADLMD-ISEEMKEAIDLSMTNIEKFHAAQLP 521
7630171_S.pombe                 458  PIIEDVKSRGEASLIDYASKFEKVQ---LKSAVLKAPFDDDLMK-ISPMIKEDIDIAFNNIFAFHSSQLR 523
N.mengitidis_MC58               273  DICADVQKRGDAALIEYTNKFDQTNAKSIDDLILTQADLNAAFERIPNDVQTALQTAARRVESYHQRQKM 342
15606967_A.aeolicus VF5         242  EILKRVREEGDRAVIEFTKKFDGVELT-PENMEVPFEELEKAYEEIEPEVREALEFAENRIRVFHEKQLE 310
16080539_B.subtilis             240  SIIEDVKANGDQAVRSYTAKFDCIEIDSP---LVTKEEFEEAYTSLDSRLLQVIRQAIENIREYHERQLQ 306
15805759_D.radiodurans R1       264  RILLDVRARGDDALRDWTERLDGPR---PAELEVP--AAELEAAQVAPELHAAIRLAAERVRAFYRQQPA 328
16129967_E.coli K12             241  DILDNVKARGDEALREYSAKFDKTTV--TALKVSAEEIAAASER-LSDELKQAMAVAVKNIETFHTAQKL 307
39996631_G.sulfurreducens PCA   268  DIIADVRARGDEALLEYTRRFDRLEADSVAALQVTEDEIEYAFAKVKDEEIAALKLAVERVARFHEKQKQ 337
32265948_H.hepaticus ATCC 5144  266  ELLDEIRTEGLDALKKHIARFDKWEVKSFEDLRISPKECLNAYNQLSSELKSALHLAYDRIYAFHRKQKM 335
15673197_L.lactis subsp. lacti  247  QIVEDIQKSGDTALFNYAKKFDGYDVNTSN-LLVTPMERETGLEQIDEDYFRILRRTKSQIEEFHKHQLG 315
15841059_M.tuberculosis CDC155  245  PIVAAVAERGAEAALDFGASFDGVR---PHAIRVPDAALDAALAGLDCDVCEALQVMVERTRAVHSGQRR 311
17230755_N.sp. PCC 7120         251  DILAHVKARGDAAVQEYTSRFDHYRPH-SHHL--SAAFIAEQAAKCSDEVKAALELAAERISSFHQKQLP 317
15925662_S.aureus subsp. aureu  237  DICQEVKVHGDKALKMYNLTFDHTKTDHL---EISHEQIKAAFDTLDEKTKQALQQSYERIKAYQESIKQ 303
16332335_S.sp. PCC 6803         265  EVIEQVKMEGDAGVLHYSRQFDFAGAT-AENLRVSEAEFAEAEKLVDPELRRAVEHAFRNIEKVHAGQMP 333
15643793_T.maritima MSB8        229  EIIRRVKEEGDRALEEFLKRFEKHPVG-IENLRVTEKEISEA--QVEEEFVETIKIVIEDLKEFHRRQEE 295
46199382_T.thermophilus HB27    243  GILEAVREEGDEALDRFSRDLDGYP---VEEVPKR--AWREAYEDLDEDLRDALETARERIEAFYREEAR 307
34556543_W.succinogenes DSM 17  267  GLIKEIRTQGDAALLAHVAKFDRWNPKSAMELKIDPSLMKRAYEGLEASLREALHSAYNRIHSFHSKQKP 336
15669621_M.jannaschii DSM 2661  260  EILKDVKEKGDEALKYYTKKFDGV--E-IEDFKVTDEEIEEAYNSVDYKVVEAIERAKENIYFFHKKQME 326
15678273_M.thermautotrophicus   267  DIISMVRDGGDDALRELTGRFDGV--T-VENFRVSREEIEEAHKNLEPGVKEALREAASNIEEFHRMQMP 333
20094973_M.kandleri AV19        273  RIFEDVVERGDEALLEYTERFDGVKLE-AEDLRVSEDDFEVARELVDERTVEALEEAAHRIEEFHRKTLP 341
20089786_M.acetivorans str. C2  260  SVLSDVRVRGDAALREYTKKFDKV--E-LAGFEVSEAEFEEALSGVGPELLEHLKVAAANIRVFHEAQLP 326
18312308_P.aerophilum str. IM2  239  KIVDDVQSGGLKAALEYSKRLDGV---------APEPHLVTPRQGGDPEVVSAALAAKSLEALYSRISP 299
15897517_S.solfataricus P2      205  DIIESVKTKGDNALYELTEKLDKVK---IDNIKAREEELKTQASKLDPKVKQAIDTAYEQLKAFHEMLVP 271
```

```
                                                640       650       660       670       680       690       700
                                       ....|....|....|....|....|....|....|....|....|....|....|....|....|
10177677_A.thaliana              379   -----------------------TEKSVENMKGVRCKRVSRSIGSVGLYVPGGTAVLPSTALMLAIPAQI 426
34904356_O.sativa (japonica cu   404   -----------------------PEKTVENMKGVRCKRITRCIGSVGLYVPGGTAVLPSTALMLAVPAQI 451
10383761_S.cerevisiae            456   ---------------------T-ETLEVETQPGVLCSRFPRPIEKVGLYIPGGTAILPSTALMLGVPAQV 504
38567265_N.crassa                531   ---------------------EEKPLQVETMPGVVCSRFSRPIEAVGCYIPGGTAVLPSTALMLGVPAMV 580
50285163_C.glabrata              459   ---------------------T-EDLEVETQPGVLCSRFPRPIEKVGLYIPGGTAVLPSTALMLGVPAKV 507
42547615_G.zeae PH1              515   ---------------------EEKSLQVETMPGIVCSRFSRPIERVGLYIPGGTAVLPSTALMLGVPAMV 564
50304609_K.lactis                452   ---------------------S-ETLDVETQPGVVCSRFPRPIEKVGLYIPGGTAILPSTALMLGVPAQV 500
38109852_M.grisea 7015           524   ---------------------EDKPLRVETMPGVVCSRFSRPIERVGLYVPGGTAVLPSTALMLGVPAMV 573
40746471_A.nidulans FGSC A4      527   ---------------------SNDALSMETMPGVVCSRFSRPIERVGCYIPGGTAVLPSTAMMLGVPAMV 576
50258877_C.neoformans var. neo   514   ---------------------EKEPLVVETMPGVTCSRFARPIARVGVYVPGGTAILPSTAIMLGVPAQV 563
44985362_A.gossypii ATCC 10895   463   ---------------------D-DILKVETQPGVVCTRFPRPIEKVGLYVPGGTAVLPSTAVMLGVPAQV 511
46099735_U.maydis 521            539   TFGNKSAGATGSGSADGSEGAQDGVLEVETMPGVVCRRFARPIESVGLYVPGGSAVLPSTALMLGVPAQV 608
31095443_H.cylindrosporum        505   ---------------------SDSTLVVETMPGVVCSRFARAIARVGLYVPGGTAILPSTALMLGIPAQV 554
49645975_Y.lipolytica CLIB99     518   ---------------------T-TELVVETQPGVVCKRFSRPIESVGLYIPGGTAVLPSTSLHLGVPAMV 566
3203_P.pastoris                  508   -----------------------ETLQVETCPGVVCSRFARPIEKVGLYIPGGTAILPSTSLMLGVPAKV 555
3757752_C.albicans               497   ---------------------KEKVMTVETSPGVYCSRFAKPIENVGLYVPGGTAVLPSTAMMLGVPAKV 546
49656425_D.hansenii CBS767       521   ---------------------KEKIMTVETAPGVYCSRFAKAIENVGLYVPGGTAVLPSTAMMLGVPAKV 570
7630171_S.pombe                  523   ---------------------P--TIAVQTMRGVVCQRMSRPINRVGLYIPGGTAVLPSTALMLGVPAKV 570
N.mengitidis_MC58                343   ES----------------------WSYTDED-GTLLGQQITPLDRVGIYVPGGKAAYPSSVIMNAMPAHV 389
15606967_A.aeolicus VF5          311   NS----------------------FFKEEE--GIILGQKVVPLEKVGVYVPGGKAAYPSTVLMNVVPASV 356
16080539_B.subtilis              307   SS----------------------WFYHRKD-GTMLGQKVTALDSAGVYVPGGTAAYPSSVLMNVIPALV 353
15805759_D.radiodurans R1        329   HG----------------------FLEHG--PDGALGQLVRPLGRVGVYVPGGLAPLISTLMHTAVPAQV 374
16129967_E.coli K12              308   P-----------------------PVDVETQPGVRCQQVTRPVASVGLYIPGGSAPLFSTVLMLATPASI 354
39996631_G.sulfurreducens PCA    338   ET----------------------WLSTTEP-DILLGQMVTPLERVGIYVPGGKASYPSSVIMNAVPARV 384
32265948_H.hepaticus ATCC 5144   336   QS----------------------WIDCEEN-GNILGSKFTPMERAGLYIPGGKAAYPSSLLMNAIPAIV 382
15673197_L.lactis subsp. lacti   316   NS----------------------WNIFKEN-GVIMGQIARPLERVALYVPGGTAAYPSTVIMNAVPALL 362
15841059_M.tuberculosis CDC155   312   TD----------------------VTTTLG-PGATVTERWVPVERVGLYVPGGNAVYPSSVVMNVVPAQA 358
17230755_N.sp. PCC 7120          318   QD----------------------IGYTDTA-GVKLGLNWVALSQVGIYVPGGRASYPSSVLMNALPAKI 364
15925662_S.aureus subsp. aureu   304   T-------------------------NQQLEE-SVECYEIYHPLESVGIYVPGGKASYPSTVLMTATLAQV 348
16332335_S.sp. PCC 6803          334   PP----------------------MHLAEIEPGVFAGEKITPLPTVGLYVPRGKGAFPSMMLMLAVPARV 381
15643793_T.maritima MSB8         296   RS----------------------FFFTTKG-GSFLGEMVVPLESVGIYVPGGKVPYFSTLLMCAVPAIV 342
46199382_T.thermophilus HB27     308   GG----------------------FLRAE--GGGVLAQLVRPLERVGVYVPGGSAPLLSTLLMTVVPAKV 353
34556543_W.succinogenes DSM 17   337   QS----------------------WLDFEEN-GTILGQKVTPMDRAGLYIPGGKAAYPSSLLMNAIPAIV 383
15669621_M.jannaschii DSM 2661   327   QI----------------------KDLNVENNGIILGQVVRAIEKVGCYVPGGRAFYPSTVLMTTIPAKV 374
15678273_M.thermautotrophicus    334   SG----------------------WMSEVR-PGVMAGQLVRPIDSVGCYIPGGRAVYPSTILMTVIPARI 380
20094973_M.kandleri AV19         342   RV----------------------DRITFDVEGTECGLTLRPIPRVGCYVPGGRAAYPSTALMTVIPARV 389
20089786_M.acetivorans str. C2   327   ET----------------------TWFMEVQPGVVLGQKATALESVGAYAPGGRASYPSTVLMTVIPARV 374
18312308_P.aerophilum str. IM2   300   PA----------------------AVDFYG--GILRQILWKPVRRAALYVPAR---YISTLVMLAVPARL 342
15897517_S.solfataricus P2       272   PN----------------------IGGGYQ--GISFGVIWRSIEKIGIYVPSGKYSYPSTLLMAGIPAKV 317
```

```
                                          710       720       730       740       750       760       770
                                     ....|....|....|....|....|....|....|....|....|....|....|....|....|....|
10177677_A.thaliana            427   AGCKTVVLATPPSK--DGSICKEVLYCAKRAGVTHILKAGGAQAIAAMAWG-----TDSCPKVEKIFGPG 489
34904356_O.sativa (japonica cu 452   AGCKTVVLATPPSR--DGSICKEVLYCAKKAGVTHVLKAGGAQ-------------------VEKIFGPG 500
10383761_S.cerevisiae          505   AQCKEIVFASPPRKS-DGKVSPEVVYVAEKVGASKIVLAGGAQAVAAMAYG-----TETIPKVDKILGPG 568
38567265_N.crassa              581   AGCNKIVFASPPRA--DGTITPEIVYVAHKVGAESIVLAGGAQAVAAMAYG-----TESITKVDKILGPG 643
50285163_C.glabrata            508   AGCKEIVFASPPRKS-DGRVSPEVVYVAEKVGASKIVLAGGAQAVAAMAYG-----TESVPKVDKILGPG 571
42547615_G.zeae PH1            565   AGCQKIVFASPPRS--DGRITPEIVYVAHKVGAESIVLAGGAQAVAALAYG-----TESVTKVDKILGPG 627
50304609_K.lactis              501   AGCKEIVFASPPRKS-DGRVSPEVVYVASKVGASKIVLAGGAQAIAAMAYG-----TESVPKVDKILGPG 564
38109852_M.grisea 7015         574   AGCQRIVLASPPRQ--DGTVTPEIVYVAHKVGAESIVLAGGAQAVAAMAYG-----TESVTKVDKILGPG 636
40746471_A.nidulans FGSC A4    577   AGCKKIVFASPPRA--DGSITPEIVYVAHKVGAESIVLAGGAQAVAAMAYG-----TESVSKVDKILGPG 639
50258877_C.neoformans var. neo 564   AGCKTIVLATPPRQ--DGSISPEVLYVAKLTGVTCILKAGGAQAVGAMAYG-----TDEVPKVDKIFGPG 626
44985362_A.gossypii ATCC 10895 512   AGCKEIVIASPPRKS-DGKISPEVVYVASKIGASKIVLAGGAQAVAALAYG-----THSVPKVDKILGPG 575
46099735_U.maydis 521          609   ARCPTVVIATPPRA--DGSISPEVLYCASKVNASAILCAGGAQAVAALAYG-----TESCPKVDKIVGPG 671
31095443_H.cylindrosporum      555   AGCKEIVLATPPRS--DGSISPEVMYVAHLVGASAILKAGGAQAVAAMAYG-----TKTVPKVDKIFGPG 617
49645975_Y.lipolytica CLIB99   567   AGCKNIVLASPPRK--DGTLSPEVVYVAHQIGAKAVVLAGGAQAVAAMAYG-----TESVPKVDKIMGPG 629
3203_P.pastoris                556   AGCKEIVFASPPKK--DGTLTPEVIYVAHKVGAKCIVLAGGAQAVAAMAYG-----TETVPKCDKIFGPG 618
3757752_C.albicans             547   AGCKNIIVASPPSRA-TGKLTPEVVYVAHKLGAKCIVMAGGAQAVTAMAYG-----TESVLKCDKILGPG 610
49656425_D.hansenii CBS767     571   AGCSNIIVATPPSRS-TGKLTPEVVYVAHKLGAKCIVMAGGAQAVTAMAYG-----TESVLKCDKILGPG 634
7630171_S.pombe                571   AGCPHVVISTPVRK--DGTVAPEIVYIANKIGAEAIILAGGAQAIAAMAYG-----ISGVPKVNKIFGPG 633
N.mengitidis_MC58              390   AGVKEIIMVV-PTP--KGERNDIVLAAAYVAGVTKVFTVGGAQAVAALAYG-----TETIPQVDKITGPG 451
15606967_A.aeolicus VF5        357   AGVEEIIMVS-PKP------NKYTLAAAYIAGVSRVFQVGGAQAIGALAYG-----TEKIPKVDKIVGPG 414
16080539_B.subtilis            354   AGVERIVLVTPPGK--DGLLSPGVLVAAAELGIKDIYKMGGAQAIAALAYG-----TETIEPVDKITGPG 416
15805759_D.radiodurans R1      375   AGVPDIVVTTPPGK--DGQVHPAILVAARELGLSRVFKVGGAQAIAALAYG-----TASVPAVDKIAGPG 437
16129967_E.coli K12            355   AGCKKVVLCSPP------PIADEILYAAQLCGVQDVFNVGGAQAIAALAFG-----TESVPKVDKIFGPG 413
39996631_G.sulfurreducens PCA  385   AGVGEIVMVA-PTP--GGEINPHVLVAARLSGVDRIFRMGGAQAVAALAYG-----TATVPRVDKITGPG 446
32265948_H.hepaticus ATCC 5144 383   AGVKEIVVCS-PTP--HNQANPLVLAALHLCGITEVYKVGGASAIGLMAYG-----CKEIPKVDVITGPG 444
15673197_L.lactis subsp. lacti 363   AGVKEIIMITPVKT--DGKVNPNILAAAEVCGIETIYKVGGAQGVAAVAYG-----TESIPKVDKIVGPG 425
15841059_M.tuberculosis CDC155 359   AGVDSLVVASPPQAQWDGMPHPTILAAARLLGVDEVWAVGGAQAVALLAYGGTDTDGAALTPVDMITGPG 428
17230755_N.sp. PCC 7120        365   AGVERIVMTVPMP---HGEINPAVLAAAQVAGVTEIYSIGGAQAVGALAYG-----TETITPVDKIVGPG 426
15925662_S.aureus subsp. aureu 349   AGVENIVVVTPPQP--NG-VSQEVLAACYITQVNQVFQVGGAQSIAALTYG-----TETIPKVDKIVGPG 410
16332335_S.sp. PCC 6803        382   AGVKKIVVCTPPDK--EGKVEPVSLVTARMAGVDEVYKLGGVQALAAIAYG-----TKTVSKVDKLIGPC 444
15643793_T.maritima MSB8       343   AGVERIAVTTPPNE--NGGISPYILKTCEILGLKEIYRMGGAHAVAALTYG-----TETVKPVDKIVGPG 405
46199382_T.thermophilus HB27   354   AGVREVIVASPP------KVHPGVLAAAWVAGADRLFAMGGAQAIAALAYG-----TGRVPRVDKIVGPG 412
34556543_W.succinogenes DSM 17 384   AGVKEIVVCT-PTP--ENEPNELLLAACHLCGIKEVYKVGGASAIAAMAYG-----TESLGRVDVITGPG 445
15669621_M.jannaschii DSM 2661 375   AGCEEIYITSPPTK--DGKGNPATLIAGDIVGVSAIYKVGGVQAIGALAYG-----TETIPKVDIIVGPG 437
15678273_M.thermautotrophicus  381   AGVERIVCCTPPAQ--DGSVPDAVLVAADMAGASEIYRVGGAQAVAAMAYG-----TETIRPVDKIVGPG 443
20094973_M.kandleri AV19       390   AGCREVVVCTPPADN-DVRASPEVLVAVEIAGADAVYRVGGAQAIAALAAG-----TETVLRVDKIVGPG 453
20089786_M.acetivorans str. C2 375   AGVKQVIVCTPPRA--DGSIHPLTLAAAKVAGADKVFKLGGVQAVGAMAYG-----TETVPKVDKIVGPG 437
18312308_P.aerophilum str. IM2 343   AGVEEVYVVTPP-----RGVSEELLAVAKELGVKAVLALGGPHGLAYAVFH-----MG----VDVVAGPG 398
15897517_S.solfataricus P2     318   AKVKEIYVASPPTQ--EGTVNPALAYVAIKLGVNEVYKIGGAQAIAALAFG-----TESVKKVYKIVGPG 380
```

```
                                              780       790       800       810       820       830       840
                                      ....|....|....|....|....|....|....|....|....|....|....|....|....|....|
10177677_A.thaliana             490 NQYVTAAKMILQN-SEAMVSIDMPAGPSEVLVIADEHASPVYIAADLLSQAEHGPDSQVVLVVVGDS-VD 557
34904356_O.sativa (japonica cu  501 NQYVTAAKMILQN-SEAMVSIDMPAGPSEVLVIADKYANPVHVAADLLSQAEHGPDSQVVLVVAGDG-VD 568
10383761_S.cerevisiae           569 NQFVTAAKMYVQNDTQALCSIDMPAGPSEVLVIADEDADVDFVASDLLSQAEHGIDSQVILVGVNLSEKK 638
38567265_N.crassa               644 NQFVTAAKMFVSNDTNAAVGIDMPAGPSEVLVIADKDANPAFVASDLLSQAEHGVDSQVILIAIDLDEEH 713
50285163_C.glabrata             572 NQFVTAAKMYVQNDTQALCSIDMPAGPSEVLVIADNNADVDFVASDLLSQAEHGIDSQVILVGVELTDDK 641
42547615_G.zeae PH1             628 NQFVTAAKMHVSNDTNAGVGIDMPAGPSEVLVVADKDANPAFVASDLLSQAEHGVDSQVILIAVDLSEQE 697
50304609_K.lactis               565 NQFVTAAKMYVQNDTQALCSIDMPAGPSEVLVICDEEADVDFVASDLLSQAEHGIDSQVILVGVSLSDSK 634
38109852_M.grisea 7015          637 NQFVTAAKMLVSNDTNAGVGIDMPAGPSEVLVIADCDANPAFVASDLLSQAEHGVDSQVVLIAVDLDEAG 706
40746471_A.nidulans FGSC A4     640 NQFVTAAKMLVSNDTSAGVSIDMPAGPSEVLVIADKAANPAFVASDLLSQAEHGVDSQVILIAIDLNEQE 709
50258877_C.neoformans var. neo  627 NQWVTAAKMLVQNDTDALVAIDMPAGPSEVLVIADYTANPVFVASDLLSQAEHGVDSQVILLAINLTPEH 696
44985362_A.gossypii ATCC 10895  576 NQFVTAAKMYVQNDMQALCAIDMPAGPSEVLVICDESADADFVAADLLSQAEHGADSQVILIGANISDEK 645
46099735_U.maydis 521           672 NQFVTAAKMLVQNETDAIVSIDMPAGPSEVLVIADREADAAFVASDLLSQAEHGPDSQVVLVGIALSSSE 741
31095443_H.cylindrosporum       618 NQWVTAAKMLVQNDTDALVSIDMPAGPSEVLVIADHTANPASSRQNLLSQAEHGVDSQVVLVAINLSAEH 687
49645975_Y.lipolytica CLIB99    630 NQFVTAAKMYVSNDTTAKVAIDMPAGPSEVLVICDKHANPAFVASDLLSQAEHGVDSQVILLAVDCDQKH 699
3203_P.pastoris                 619 NQFVTAAKMMVQNDTSALCSIDMPAGPSEVLVIADKYADPDFVASDLLSQAEHGIDSQVILLAVDMTDKE 688
3757752_C.albicans              611 NQFVTAAKMYVQNDTQALCSIDMPAGPSEVLVIADSNADADFVASDLLSQAEHGVDSQVILIGVGLSDEK 680
49656425_D.hansenii CBS767      635 NQFVTAAKMHVQNDTQALCSIDMPAGPSEVLVIADSEADADFVASDLLSQAEHGVDSQVILVGVGLTKEK 704
7630171_S.pombe                 634 NQFVTAAKMHVQNDYGALVAIDLPAGPSEVLVIADETCNPESVALDLLSQAEHGLDSQIILLTVSLSPEM 703
N.mengitidis_MC58               452 NAFVAAAKRRVF----GVVGIDMVAGPSEILVIADGTTPADWVAMDLFSQAEHDEIAQAILIGTSQAYLD 517
15606967_A.aeolicus VF5         415 NIYVALAKKLVF----GTVDIDMIAGPSEVLVIADERANPTWVAADMLSQAEHDELAASILLTPSEELAN 480
16080539_B.subtilis             417 NIYVALAKREVF----GDVDIDMIAGPSEIVVLADETAIPSEIAADLLSQAEHDKLSSCVFVTDSMALAE 482
15805759_D.radiodurans R1       438 NLFVVIAKRLVY----GQTGIESLPGPTETLVVADDSASPRYVAADLLAQAEHN-GAEPVLVSVSRELLL 502
16129967_E.coli K12             414 NAFVTEAKRQVSQR-LDGAAIDMPAGPSEVLVIADSGATPDFVASDLLSQAEHGPDSQVILLTPAADMAR 482
39996631_G.sulfurreducens PCA   447 NIYVATAKKLVF----GQVGIDMIAGPSEILVINDGSGTPAHIAADLLSQAEHDELASSILITTDRGFGE 512
32265948_H.hepaticus ATCC 5144  445 NIFVACAKKLVF----GEVNIDMVAGPSEIAIIADSQANPLYIAYDLLSQAEHDEMASSILISDSAPLIE 510
15673197_L.lactis subsp. lacti  426 NIFVATAKKICY----GVVDIDMIAGPSEVLVIADETANPKYIAADLMAQAEHDKLASAILVTTSEKLVQ 491
15841059_M.tuberculosis CDC155  429 NIYVTAAKRLCR----SRVGIDAEAGPTEIAILADHTADPVHVAADLISQAEHDELAASVLVTPSEDLAD 494
17230755_N.sp. PCC 7120         427 NAYVAEAKRQVF----GTVGIDSIAGPSEILVVADRQNNPEWIAWDLLSQAEHDPSAQSILITDSESFAQ 492
15925662_S.aureus subsp. aureu  411 NQFVAYAKKYLF----GQVGIDQIAGPTEIALIIDDTADLDAIVYDVFAQAEHDELARTYVIGEDAQVLK 476
16332335_S.sp. PCC 6803         445 SIYGAAAKRLLS----GIVDVGLPAGPSESIVLADETTDPKLAALDLLIEAEHGSDSAALLVTHSASLAE 510
15643793_T.maritima MSB8        406 GVFVTLAKKHVY----GDVGIDSIAGPSEIAIVTDGSADLDLIAADFLSQAEHDENAMSVVITTSKEVFE 471
46199382_T.thermophilus HB27    413 NRYVVAAKRLVY----GTVGIDGLAGPTETMIIADGSASPRLLAADLLAQAEHGPDSEPWLLSPDRALLE 478
34556543_W.succinogenes DSM 17  446 NIYVATAKKLVF----GQVNIDMVAGPSEIGILADESAKAPWVALDLLSQAEHDEMASSILVTPSVELAD 511
15669621_M.jannaschii DSM 2661  438 NIYVTTAKKMVY----GEVAIDFLAGPSEVLIIADETANAEFVALDFIAQAEHDPNASCVITTTSEKKAE 503
15678273_M.thermautotrophicus   444 NIFVTAAKKLVY----GEVDIDFPAGPSEVLIIADETASPEYIALEILAQAEHDPQAASVLVTDSRDLAL 509
20094973_M.kandleri AV19        454 NVYVTAAKLLAYS--RGLTDVDMPAGPSEVFVIADDSANPDWLARDLIAQAEHDPHAAAVLATDSEEIAR 521
20089786_M.acetivorans str. C2  438 NVFVTSAKMQVR----NVAEIDFPAGPSEVLIIADDSADAAMVASDIIAQAEHDPNAVSVLVTTSEILAE 503
18312308_P.aerophilum str. IM2  399 GLYVQAAKYILS----QYVGIDGIEGPTELVIYAEG-VPPEVAVRGALAQLEHGPTSFAYLLSPDGELLK 463
15897517_S.solfataricus P2      381 NVYVQAAKYLVS----SVVGIDGIEGPTELVIIADETAKAEYVALDMKAQAEHGPDTYIVLLSNDDELIR 446
```

```
                                                      850       860       870       880       890       900       910
                                                ....|....|....|....|....|....|....|....|....|....|....|....|....|....|
10177677_A.thaliana                 558   LNAIEEEIAKQCKSLPRGEFASKALSHSF-TVFARDMIEAISFSNLYAPEHLIINVKDAEKWE-GLIENA 625
34904356_O.sativa (japonica cu      569   LGAIEAEVSKQCSALPRGEFASKALGHSF-TVFAKDMVEAISFSNMYAPEHLIINVKDAEQWE-DLVENA 636
10383761_S.cerevisiae               639   IQEIQDAVHNQALQLPRVDIVRKCIAHST-IVLCDGYEEALEMSNQYAPEHLILQIANANDYV-KLVDNA 706
38567265_N.crassa                   714   LQAIEDEVHRQATELPRVQIVRGSIAHSI-TVQVKTVEEAMELSNKYAPEHLILQIKEAEKAV-DLVMNA 781
50285163_C.glabrata                 642   IQQIQDAVHRQALELPRVDIVRKCIAHSS-IIVCDSYQSAFDMSNRYAPEHLILQIENAKDYV-KLVDHA 709
42547615_G.zeae PH1                 698   LQAIEDEVHNQAVALPRVDIVRGSIAHSV-TVQVKDITDAMRISNDYAPEHLILQIKDAEKAV-DQVMNA 765
50304609_K.lactis                   635   IEALQNAVHEQAMQLPRVDIVRKCIAHSS-IILCDSYEEAFKMSNQYAPEHLILQISNAEDYV-KDVDHA 702
38109852_M.grisea 7015              707   LKAIEDEVHRQAMALPRVDIVRGSIKHSI-TISVRNIEEAMRISNDYAPEHLILQLKNAEAVV-DMVMNA 774
40746471_A.nidulans FGSC A4         710   LKAIEDEVDRHARALPRMDIVRGSLAHSV-TFVVRDLDEAMALSNDYAPEHLILQIQNAEAAV-EKVQNA 777
50258877_C.neoformans var. neo      697   LAAIEAEIDRQARALPRVKIAREAIKKSV-TVEVKDLEEAVKFSNEYAPEHLILHLEKAEEVV-AEIENA 764
44985362_A.gossypii ATCC 10895      646   VHSIQAAVHHQAISLPRVDIIRKSIAHSS-IILSSTAEEAFALSNRYAPEHLILHLENASEYL-PLVDHA 713
46099735_U.maydis 521               742   LAAIEAEVDRQARALPRVDVVRQAIDKSV-TVIVPDRATAMEWSNAYAPEHLILQTRDAESLV-ECVQNA 809
31095443_H.cylindrosporum           688   LASIEREVDEQAHALSRVDIVRQSIVKSI-IVKVSSVDEALAFSNDYAPEHLILHLEKPSEKV-AMVDNA 755
49645975_Y.lipolytica CLIB99        700   LDAIDEELHAQALALPRVDIIRKCIAHSS-ALSVKTMDEAFKLSNQYAPEHLILQIEDADKYV-PLVDHA 767
3203_P.pastoris                     689   LARIEDAVHNQAVQLPRVEIVRKCIAHST-TLSVATYEQALEMSNQYAPEHLILQIENASSYV-DQVQHA 756
3757752_C.albicans                  681   LNEFQAAVERQAKVLPRKDIVAKCLAHSY-ILLAKTYKEAFDLSNQYAPEHLILQIDDAPSYVPDSIENA 749
49656425_D.hansenii CBS767          705   LNEFESAVKKQAQVLPRKDIVAKCLSHSY-TLLVDTYDEAFKLSNKYAPEHLILQVENARSFVPDYIENA 773
7630171_S.pombe                     704   FDRIQKAINDHALRLSRSYIIKHAIKKSV-IVQVDNVDQAFEWSNLYGPEHLVLHLKNASSYI-PKIDNA 771
N.mengitidis_MC58                   518   EVEAAMDRLIET--MPRRDIIEASLGNRGAMILAKDLDEACEIANYISPEHLELSVENPQEWA-KKIRHA 584
15606967_A.aeolicus VF5             481   KVKEEVERLLS--QLERKEIAQKSLEKFGTIFLVKDLYHACEVANYIAPEHLEVMVREPM-ALLPELKHA 547
16080539_B.subtilis                 483   TVSAEVNKQLET--LPRREIAEASVRDYGCIYVAETMVEAIETVNTLAPEHLEIIT-QSPEALLGSIKHA 549
15805759_D.radiodurans R1           503   AVQAELNEQLENLPEPNRSWARDSVGARMKVVLADSLDEALDLANLYAPEHLCLLT-RDPWSLLGQVRRA 571
16129967_E.coli K12                 482   --RVAEAVERQLAELPRAETARQALNASR-LIVTKDLAQCVEISNQYGPEHLIIQTRNARELV-DSITSA 548
39996631_G.sulfurreducens PCA       513   QVATEVE--RQLAQLSRETIARTSWETYGAVIVAGSLDEAIAFSNRIAPEHLELAVAN-PFEILPRIKNA 579
32265948_H.hepaticus ATCC 5144      511   SVQKHIE--QILPSMPRAEIAGASIKNRAVMIYTRNLQESIEIANAIAPEHLEVLT-TTPFDTLPYIKHA 577
15673197_L.lactis subsp. lacti      492   QVDEELNRQVQNLE--RSEIIESYISNYGGAIVVKNIDEAFDVSNQLAPEHLEVLT-SEPLTQLPKIKNA 558
15841059_M.tuberculosis CDC155      495   ATDAELAGQLQTTVHRERVTAAL-TGRQSAIVLVDDVDAAVLVVNAYAAEHLEIQTADAPQ-VASRIRSA 562
17230755_N.sp. PCC 7120             493   QVIAAVE--QILTTLPSQKVASSSWQNHGAVIIVRDLAESIPLLNQLAPEHVELCVDN-PQLLASQIKCA 559
15925662_S.aureus subsp. aureu      477   DLESRIAKALPNVD--RYDIVSKSIANQHYLIHASNFDEACHVMNTIAPEHASIQTVN-PQPYIEKVKYV 543
16332335_S.sp. PCC 6803             511   KALGYLGEYLEKLPPWRKKFCEDGLGSYGGILLTDSLQASLDFINDYAPEHLQVLTAD-PLKLVGKIDNA 579
15643793_T.maritima MSB8            472   KLPQVIERHLEALPEERRKTARISTENFGTIILTDSLKRAFEISNLIAPEHLEVLVEN-PFEPLGHIKNA 540
46199382_T.thermophilus HB27        479   RVEAELSRQLQDLPRAEVARQALEKGG---LVLTKDLEEAFALANLYAPEHLSLALSD-PLPWLEKVQNA 544
34556543_W.succinogenes DSM 17      512   AVDVEVERALERLD--RKEISSKSIYTRGAIIIAKDMNEAVSLMNEIAPEHLEVLVEN-PFGWLPEIRHA 578
15669621_M.jannaschii DSM 2661      504   EIKNKIFEEIEK-AE-RKEIILKALENS--AILIGDLEECIEFSNKYAPEHLEILTKN-PEEVLNKIKHA 568
15678273_M.thermautotrophicus       510   EVKEMVHENIKY-ME-RANIIRESLERYGMIVLTADIDEAVDFSNAYAPEHLVIMTDS-PEETLEGIRNA 576
20094973_M.kandleri AV19            522   AVKERVEELLDAGIE-REEIVLKALDRNGWIVVLDSLEECVRLANRYAPEHLQLCVEN-PEELLQDVENA 589
20089786_M.acetivorans str. C2      504   AVRQEVLLQAEN-TA-RSEIVKTSLENAA-VLISDTLEQSIDFSNKFAPEHLEIMVED-SDFVLNRIKNA 569
18312308_P.aerophilum str. IM2      464   KAEELYVRERTSSMG-PLKVKK-----------VGGIDEAVSFIDEIAPEHLEVWG-RRE--VAYRVRNV 518
15897517_S.solfataricus P2          447   RVEEKIKNDKK-----IYYIIK-----------TKNLDEAIEIANKIAPEHLSLYV-KDAYTLMDKIVNA 499
```

```
                                              920       930       940       950       960       970       980
                                       ....|....|....|....|....|....|....|....|....|....|....|....|....|....|
10177677_A.thaliana                626 GSVFIGPWTPESVGDYASGTNHVLPTYGYARMYSGVSLDSFLKFMTVQSLTEEGLRNLGPYVATMAEIEG 695
34904356_O.sativa (japonica cu     637 GSVFLGQWTPESVGDYASGTNHVLPTYGYARMYSGVSLNSFLKYITVQSLSEEGLRSLGPHVAKMAEVEG 706
10383761_S.cerevisiae              707 GSVFVGAYTPESCGDYSSGTNHTLPTYGYARQYSGANTATFQKFITAQNITPEGLENIGRAVMCVAKKEG 776
38567265_N.crassa                  782 GSVFIGAWTPESVGDYSAGVNHSLPTYGFAKQYSGVNLASFVKHITSSNLTAEGLKNVGQAVMQLAKVEE 851
50285163_C.glabrata                710 GSVFVGAYTPESCGDYSSGTNHTLPTYGYARQYSGANTSTFQKFITAQSITPEGLDNIGRAVMCVAKVEG 779
42547615_G.zeae PH1                766 GSVFIGHWTPESVGDYSAGVNHSLPTYGFAKQYSGVNLGSFQKHITSSNLTADGLKNVGTAVMQLAKVEE 835
50304609_K.lactis                  703 GSIFVGAYTPESCGDYSSGTNHTLPTYGYARQYSGVNTATFQKFITSQVVTPVGLEHIGHAVMSVAKVEG 772
38109852_M.grisea 7015             775 GSVFIGQWTPESVGDYSAGVNHSLPTYGYAKQYSGVNLGSFVKHITSSNLTADGLRNVGEAVMQLAKVEE 844
40746471_A.nidulans FGSC A4        778 GSVFIGQWTPESVGDYSAGVNHSLPTYGYAKQYSGVNLGSFLKHITSSNLTADGLLRLSKTVETLAAVEG 847
50258877_C.neoformans var. neo     765 GSVFVGPFSPESCGDYASGTNHTLPTNGFARQFSGVNTLSFQKHITSQIVSAEGLKKLGPYVIRLAEREG 834
44985362_A.gossypii ATCC 10895     714 GSVFVGAYTPESCGDYSSGTNHTLPTYGYARQYSGVNTATFQKFVTSQEIYPEGLQNIGHAVMSLAKVEG 783
46099735_U.maydis 521              810 GSIFVGPWSPESCGDYMSGTNHSLPTAAYAKSYSGVNVATFEKCITSQSLTQQGLKGFAQNVIDLAMCEG 879
31095443_H.cylindrosporum          756 GSVFVGPYTPESCGDYASGTNHTLPTNGYARQFSGVNTLSFQKHITSQEITQAGLRGLGPVVATLADCEG 825
49645975_Y.lipolytica CLIB99       768 GSIFVGAYSPESCGDYSSGTNHTLPTYGYARMYSGVNTGTFTKHITSQQLTREGLEIIGPAVMTLAATEG 837
3203_P.pastoris                    757 GSVFVGAYSPESCGDYSSGTNHTLPTYGYARQYSGVNTATFQKFITSQDVTPEGLKHIGQAVMDLAAVEG 826
3757752_C.albicans                 750 GSVFVGALSPESCGDYSSGTNHTLPTYGYARQYSGVNTATFQKFITSQEVTEKGLQNIGKAVMELARVEG 819
49656425_D.hansenii CBS767         774 GSVFVGGLSPESCGDYSSGTNHTLPTYGYARQYSGVNTATFQKFITSQDVTEEGLKSIGKAVMTLAEVEG 843
7630171_S.pombe                    772 GSVFVGPWSPVSMGDYASGTNHTLPTYGYASSYSGVSTDSFLKYITTQELTEEGIQRLGPTVIRLAELEG 841
N.mengitidis_MC58                  585 GAIFMGRYTGESLGDYCAGPNHVLPTSRTARFSSPLGTYDFQKRSSLIQVSEQGAQKLGETASVLAHGES 654
15606967_A.aeolicus VF5            548 GAIFLGDYTTEPLGDYVLGPNHTLPTGGSTRFFSPLGVYDFIKRSSVLYVSREGFKRVANQAEAIAKAEG 617
16080539_B.subtilis                550 GAIFLGRYSPEPVGDYFAGPNHVLPTNGTARFSSPLNVTDFQKKSSIISYSQSAFEEHAESIAAFARLEG 619
15805759_D.radiodurans R1          572 GGVFVGEASMEALGDYVAGPSHVMPTGGTARFMSPVNVRDFQNIISVVGVNEETLRRIGPAAATLARAEG 641
16129967_E.coli K12                549 GSVFLGDWSPESAGDYASGTNHVLPTYGYTATCSSLGLADFQKRMTVQELSKEGFSALASTIETLAAAER 618
39996631_G.sulfurreducens PCA      580 GAIFLGHFTPEAAGDYLAGPNHTLPTGGTARFFSPLSVDDFVKKSSIVYFSAAGLNRLGRDIVSIAEMEG 649
32265948_H.hepaticus ATCC 5144     578 GAIFLGEHSSEPIGDYLAGPNHTLPTGGSARFFSPLGVEHFMKKSSIIAFSATALEEVGESCALLAQSES 647
15673197_L.lactis subsp. lacti     559 GSIFIGEYTPEPLGDYMSGSNHVLPTGGTAKFYSGLGVYNFIKYLTYSYYPKEVLAAFKEDVETFAKSEG 628
15841059_M.tuberculosis CDC155     563 GAIFVGPWSPVSLGDYCAGSNHVLPTAGCARHSSGLSVQTFLRGIHVVEYTEAALKDVSGHVITLATAED 632
17230755_N.sp. PCC 7120            560 GSLFLGRYTPEAIGDYLGGPNHVLPTSRSARFASGLSVYDFLKRITYLECNQAALQKIGQSAVTLAETEG 629
15925662_S.aureus subsp. aureu     544 GALFIGHYSPEVIGDYVAGPSHVLPTNRTARFTNGLSVNDFLTRNTVIHLSKDTFEQIADSAQHIAHVEA 613
16332335_S.sp. PCC 6803            580 GEILLGNYTPSSAATYAIGVNAVLPTGGFARSYSAVSVFDFLKRSTLAYLTEEGFAGVKETVTTLADYED 649
15643793_T.maritima MSB8           541 GSVFLGKYTCESVGDYGAGPNHVLPTFRSARFSSGLRVSDFTKKIFITHLSEEDFRRKSELYSKMARWEG 610
46199382_T.thermophilus HB27       545 GGVFLGEGSPEALGDYIAGPSHVMPTSGTARFQGGLAVRDFLKVIPVVGLSEGAARELAKKGALLARAEG 614
34556543_W.succinogenes DSM 17     579 GAIFLGENTPEPIGDYIAGPNHTLPTGGTARFYSPLSTEHFMKKSSILSFSERGIRELGHHCAKLAQTEG 648
15669621_M.jannaschii DSM 2661     569 GSVFLGEYSPVPVGDYASGTNHVLPTSQFARMSSGLNVETFLKKITYQKLDKESLKNIADIVITLAEAEG 638
15678273_M.thermautotrophicus      577 GSIFLGELSPVAAGDYGSGTNHVLPTSGCARMYSGLSTESFIKKPTVQRITKEGLRNLQGTVLKLAEYEG 646
20094973_M.kandleri AV19           590 GAVFVGHLTAVPFGDYATGPNHVLPTGGFARARGALGTWDFVKIIPIQRLREGDVERLAPIVEELAEREG 659
20089786_M.acetivorans str. C2     570 GSIFVGNYAPVPVGDYASGTNHVLPTAGYARIYSGLNINHFLKYSSIQKISKSGLESLKETIIALAEEEG 639
18312308_P.aerophilum str. IM2     519 GAVSV--NMPSPYLDYVAGISHVLPTGGTARWRGVITPLAFMKPIGIAEAV--GELTLREAARKLAEYEG 584
15897517_S.solfataricus P2         500 GAISLG-NTPPAIIDYVAGPNHILPTNGWAKIRGGITVYDFIKPTMYANVRDI-NKQLLEASISLANYEG 567
```

```
                                                 990       1000
                                           ....|....|....|....|...
10177677_A.thaliana                 696   LDAHKRAVTLRLKDIEAKQLA-- 716
34904356_O.sativa (japonica cu      707   LEAHRRAVTLRLQDIEATVTV-- 727
10383761_S.cerevisiae               777   LDGHRNAVKIRMSKLGLIPKDFQ 799
38567265_N.crassa                   852   LEAHRRAVSIRLEHMSKSN---- 870
50285163_C.glabrata                 780   LDGHRNAVKIRMSKLGLLPKDFN 802
42547615_G.zeae PH1                 836   LEAHRRAVEIRLNYLKQQQ---- 854
50304609_K.lactis                   773   LDAHRNAVKIRMSKLGLLPSGFE 795
38109852_M.grisea 7015              845   LEAHRRAVSIRMEYMNKQANQ-- 865
40746471_A.nidulans FGSC A4         848   LDAHKRAVSIRVAAMKQEQL--- 867
50258877_C.neoformans var. neo      835   LEAHANAVRVRLAELNKQ----- 852
44985362_A.gossypii ATCC 10895      784   LDAHRNAVKIRMAKLKLLDADFD 806
46099735_U.maydis 521               880   LQAHAMAVKIRLDRIQS------ 896
31095443_H.cylindrosporum           826   LQAHANAVRVRLVNPQIS----- 843
49645975_Y.lipolytica CLIB99        838   LDAHRMAVKVRLDAMKKE----- 855
3203_P.pastoris                     827   LDAHRNAVKVRMEKLGLI----- 844
3757752_C.albicans                  820   LEAHRRAVEIRMERMAETK---- 838
49656425_D.hansenii CBS767          844   LEAHRNAVHVRMEKLGLL----- 861
7630171_S.pombe                     842   LTAHADAVRVRGVRL-------- 856
N.mengitidis_MC58                   655   LTAHARAAEFRMK---------- 667
15606967_A.aeolicus VF5             618   LTAHALAVKVRKND--------- 631
16080539_B.subtilis                 620   LEAHARSIEARERRISK------ 636
15805759_D.radiodurans R1           642   LEAHARAVESRLK---------- 654
16129967_E.coli K12                 619   LTAHKNAVTLRVNALKEQA---- 637
39996631_G.sulfurreducens PCA       650   LEAHGRSVSIRLK---------- 662
32265948_H.hepaticus ATCC 5144      648   LSAHAQAVLTRLDSIKSKRE--- 667
15673197_L.lactis subsp. lacti      629   LTAHANSISVRFDEM-------- 643
15841059_M.tuberculosis CDC155      633   LPAHGEAVRRRFER--------- 646
17230755_N.sp. PCC 7120             630   LPAHAGSVAVRLQGLKDM----- 647
15925662_S.aureus subsp. aureu      614   LYNHQQSILIRQS---------- 626
16332335_S.sp. PCC 6803             650   FPAHALAIRERENLL-------- 664
15643793_T.maritima MSB8            611   FEAHARAIDVRREKL-------- 625
46199382_T.thermophilus HB27        615   LEGHARSLDLRR----------- 626
34556543_W.succinogenes DSM 17      649   LDAHKESVLARLV---------- 661
15669621_M.jannaschii DSM 2661      639   LFGHAEAVRRRLK---------- 651
15678273_M.thermautotrophicus       647   LHAHAESFRRRLRD--------- 660
20094973_M.kandleri AV19            660   LPNHAEAVRARRS---------- 672
20089786_M.acetivorans str. C2      640   LQAHADAIRTRFGYKPSK----- 657
18312308_P.aerophilum str. IM2      585   FKYHGEALR-------------- 593
15897517_S.solfataricus P2          568   FIIHGKSIGARYE---------- 580
```
