# Supplementary material for: The role of gene fusions in the evolution of metabolic pathways: the histidine biosynthesis case
Source: BMC Evol Biol. 2007 Aug 16;7(Suppl 2):S4. doi: 10.1186/1471-2148-7-S2-S4 (PMC1963479; doi:10.1186/1471-2148-7-S2-S4)
Supplement: Additional file 7 — Pairwise identity values within HIS4 domains. Identity values for the pairwise comparison of the different domains composing HIS4 proteins (the standard deviation is also shown). [file 1471-2148-7-S2-S4-S7.pdf]

identity values of the pairwise comparison of all domai

	weighted sample
	**average±standard deviation**
N-terminal	0.22 ± 0.06
HisI	0.6 ± 0.08
HisE	0.57 ± 0.08
HisD	0.64 ± 0.11
